# Tea consumption and risk of incident dementia: A prospective cohort study of 377 592 UK Biobank participants

**DOI:** 10.1038/s41398-022-01923-z

**Published:** 2022-04-26

**Authors:** He-Ying Hu, Bang-Sheng Wu, Ya-Nan Ou, Ya-Hui Ma, Yu-Yuan Huang, Wei Cheng, Lan Tan, Jin-Tai Yu

**Affiliations:** 1 Department of Neurology, Qingdao Municipal Hospital, Qingdao University, Qingdao, China; 2 Department of Neurology and Institute of Neurology, Huashan Hospital, Shanghai Medical College, Fudan University, Shanghai, China; 3 Institute of Science and Technology for Brain-Inspired Intelligence, Fudan University, Shanghai, China

**Keywords:** Long-term memory, Diseases

## Abstract

As a widely consumed beverage, tea boasts diverse health benefits. Herein, we aimed to investigate the association between tea consumption and dementia risk. We conducted a prospective cohort study with 377 592 UK Biobank participants during a 9-year follow-up. Cox regression models adjusted for age, sex, ethnicity, Townsend deprivation index, education, body mass index, lifestyle factors, dietary factors and *apolipoprotein E4* status were used to examine the association of tea consumption with dementia risk. Subgroup analyses stratified by age, sex and forms of dementia (Alzheimer’s disease [AD] and vascular dementia [VD]) were performed. Moreover, the restricted cubic splines were used to calculate the nonlinear relationship between daily dosage of tea and dementia risk. After adjustment for all covariates, tea drinkers were 16% (95% confidence interval: 8–23) less likely to develop dementia compared with non-drinkers. Moderate consumption (1–6 cups/day) of tea exerted significant protective effects. Subgroup analyses showed that mid-aged participants or males benefited more from tea consumption. Moreover, moderate drinkers had a 16–19% lower hazard of AD and a 25–29% lower hazard of VD. Furthermore, a U-shaped association between tea consumption and dementia risk was shown (*P*_non-linearity_ = 7E^−04^), and the consumption of around three cups per day showed the strongest protective effect. Within 3 cups/day, drinking one extra cup of tea per day brought a 6% reduction of incidence. In conclusion, moderate consumption of tea was significantly associated with a reduced risk of dementia, suggesting that tea consumption could be a modifiable lifestyle factor for dementia.

## Introduction

Dementia is an important public health concern currently, with around 55 million cases worldwide and an incidence of more than 10 million new cases per annum [1]. Dementia has become a major cause of disability, dependency as well as mortality among older people, and has posed substantial burden on patients, their carers, families as well as the society [2, 3]. Dementia is composed of Alzheimer’s disease (AD), which may contribute to 60–70% of cases, vascular dementia (VD), which may contribute to 25% of cases, and other forms of dementia [1, 4]. The development of dementia is associated with genetic and environmental factors. Among the environmental factors, diet is a potentially modifiable lifestyle factor for preventing dementia [4–7].

Tea is a widely consumed beverage around the world and it contains various kinds of biomolecules such as polyphenols. Tea intake has been found to be associated with the prevention of various diseases, such as stroke, cardiovascular diseases and neurodegenerative diseases [8–10]. Recently, accumulating epidemiological studies and systemic reviews have suggested that tea intake can suppress brain aging and ameliorate cognitive dysfunction [11–14]. A population-based study has found that green tea can mitigate the pathological changes of AD [15]. AD mouse models showed that the main component of black tea could inhibit the deposits of amyloid in the brain and reduce Aβ pathology [16–18]. Mechanistic studies revealed that tea biomolecules (e.g., epigallocatechin gallate [EGCG], the predominant polyphenol in green tea) could invoke extensive cellular pathways of antioxidants and activities of neurorescue, which might prevent memory deficits [11, 12, 19]. Also, tea biomolecules were found to have anti-inflammatory properties, thereby halting the progression of cognitive decline [20, 21]. Tea was reported to play a neuroprotective role in the cognitive decline not only resulting from aging, but also from ischemia-reperfusion [22]. Previous studies suggested that tea had positive impacts (e.g., vasodilative functions) on cerebral blood vessels [23]. It is worth noting that coffee was found to mitigate the pathological progression of dementia as well as slow cognitive decline in recent animal studies, and tea shared some molecules with coffee [24, 25]. Taken together, it is plausible to hypothesize that tea may reduce the incidence of dementia.

The relationship between tea consumption and dementia has not been well established yet. Although most studies in healthy aging showed that tea was protective against dementia [11, 14, 26, 27], some studies reported no significant association [28]. The credibility of these findings is easily compromised by the cross-sectional designs, insufficient follow-up periods, small sample sizes or different reference groups. A recent cohort study reported that tea consumption was associated with lower risks of dementia, VD, poststroke dementia and stroke [29]. Evidence from human clinical trials suggested that moderate (1–6 cups/day) rather than excessive consumption of tea could bring diverse health benefits [8, 30]. Therefore, we performed a prospective cohort study to examine (1) the relationship between daily dosage and dementia risk; and (2) the effect modification by age, sex as well as the effects of tea consumption on separate outcomes (e.g., AD and VD) using large-scale data from 377 592 older adults from the UK Biobank (UKB).

## Methods

### Study population

This is a large-scale prospective cohort study of participants enrolled in the UKB, which recruited more than 500,000 participants aged 37–73 years between 2006 and 2010. The participants attended 1 of 22 assessment centers across the United Kingdom, and completed the touchscreen and nurse-led questionnaires. Data on clinic, gene, and risk factor were obtained at baseline. Since recruitment, the participants have been followed for clinical outcomes including dementia via hospital inpatient records, death certificates and primary care records. The UKB study received approval from the National Health Service (NHS) NorthWestMulticenter Research Ethics Committee. All participants provided written informed consent according to the Declaration of Helsinki. In this study, we excluded participants with prevalent dementia at baseline, and those with missing data on tea consumption, leaving 417 085 participants included.

### Tea consumption

Data on tea consumption, a self-reported item, were collected at baseline as part of the UKB touchscreen questionnaire, where participants were asked ‘How many cups of tea do you drink a day? (including black and green tea)’ (UKB Data-field ID:1488), with open-ended responses to the question. Participants were divided into six categories according to tea consumption, including non-drinkers as well as drinkers consuming 1–2 cups/day, 3–4 cups/day, 5–6 cups/day, 7–8 cups/day, and ≥9 cups/day.

### Dementia diagnoses

The diagnoses of all-cause dementia (ACD) were obtained using hospital inpatient records from the Hospital Episode Statistics for England, the Scottish Morbidity Record data for Scotland, and the Patient Episode Database for Wales. Additional cases were detected through linkage to death register data from NHS Digital. Diagnoses were recorded using the International Classification of Diseases (ICD-9 and ICD-10) coding system. Individuals with ACD were identified as having a diagnosis for AD (code 331.0 in ICD-9 and codes F00 and G30 in ICD-10), VD (codes 290.4 in ICD-9 and codes F01 and I67.3 in ICD-10), or other dementia classifications (codes 290, 291.2, 294.1, 331.0-331.2 and 331.5 in ICD-9; codes A81.0, F02, F03, F05.1, F10.6, G31.0, G31.1, and G31.8in ICD-10). Moreover, dementia diagnoses were retrieved from primary care data using read codes (version 2 [Read v2] and version 3 [CTV3 or Read v3]). Follow-up visits began on date of attending assessment center, and participants were followed up to the earliest incident dementia diagnosis, date of death, the last data collection date by the general practitioner, or the last time of hospital inpatient admission, whichever occurred first.

### Statistical analysis

Before analyses, individuals aged < 45 years at baseline were excluded since they had a low risk of incident dementia. Then we excluded individuals who reported tea consumption of more than 15 cups a day (4 standard deviations [SD] above the mean). Baseline sociodemographic, lifestyle, and main dietary characteristics in participants stratified by dementia status (incident dementia and no incident dementia) were summarized.

Cox proportional hazard regression models were used to examine the association of tea consumption with incident dementia, with non-consumption as reference and the duration of follow-up as the timescale (*N* = 377,592). Hazard ratios (HRs) with 95% confidence intervals (CIs) were reported for all analyses. Two models were fitted in our analyses. Model 1 was adjusted for age at baseline (continuous), sex (female/male) and ethnicity (white/non-white). In model 2, the following covariates were additionally considered: Townsend deprivation index (TDI) (combining information on social class, employment, car availability and housing; continuous); education (higher [with college/university degree or other professional qualification]/lower); body mass index (BMI) (continuous); lifestyle factors including typical sleep duration (continuous), smoking status (never/previous/current) and alcohol status (never/previous/current); dietary factors including total consumption of vegetables (continuous), fruit (continuous) and fish (continuous); *apolipoprotein E4* (*APOE4*) carrier status (carrier/non-carrier status as defined by genetic information). To investigate whether age or sex modified the effects of tea consumption on dementia, the subgroup analyses stratified by age (midlife [≤65 years old] and late-life [>65 years old]) and sex were conducted, respectively. Additionally, AD and VD were considered as separate outcomes and the subgroup analyses stratified by the form of dementia (AD/VD) were performed.

To explore the non-linear effects of tea consumption on incident dementia, the restricted cubic splines with five knots were introduced in the fully adjusted model using continuous measures of tea consumption.

Sensitivity analyses were performed after restricting participants to those with a follow-up time of ≥4 years. In addition, given that underlying dementia might change the dietary habits before diagnosis, we repeated the analyses after excluding incident dementia cases occurring during the first year of follow-up in order to limit the possibility of reverse causality. We also performed sensitivity analyses after excluding individuals with a history of stroke at baseline. *P* < 0.05 was considered to be statistically significant. R version 4.0.5 [31] and SPSS 25.0 were used for statistical analyses and figure preparation.

## Results

### Participant characteristics

**Fig. 1:**
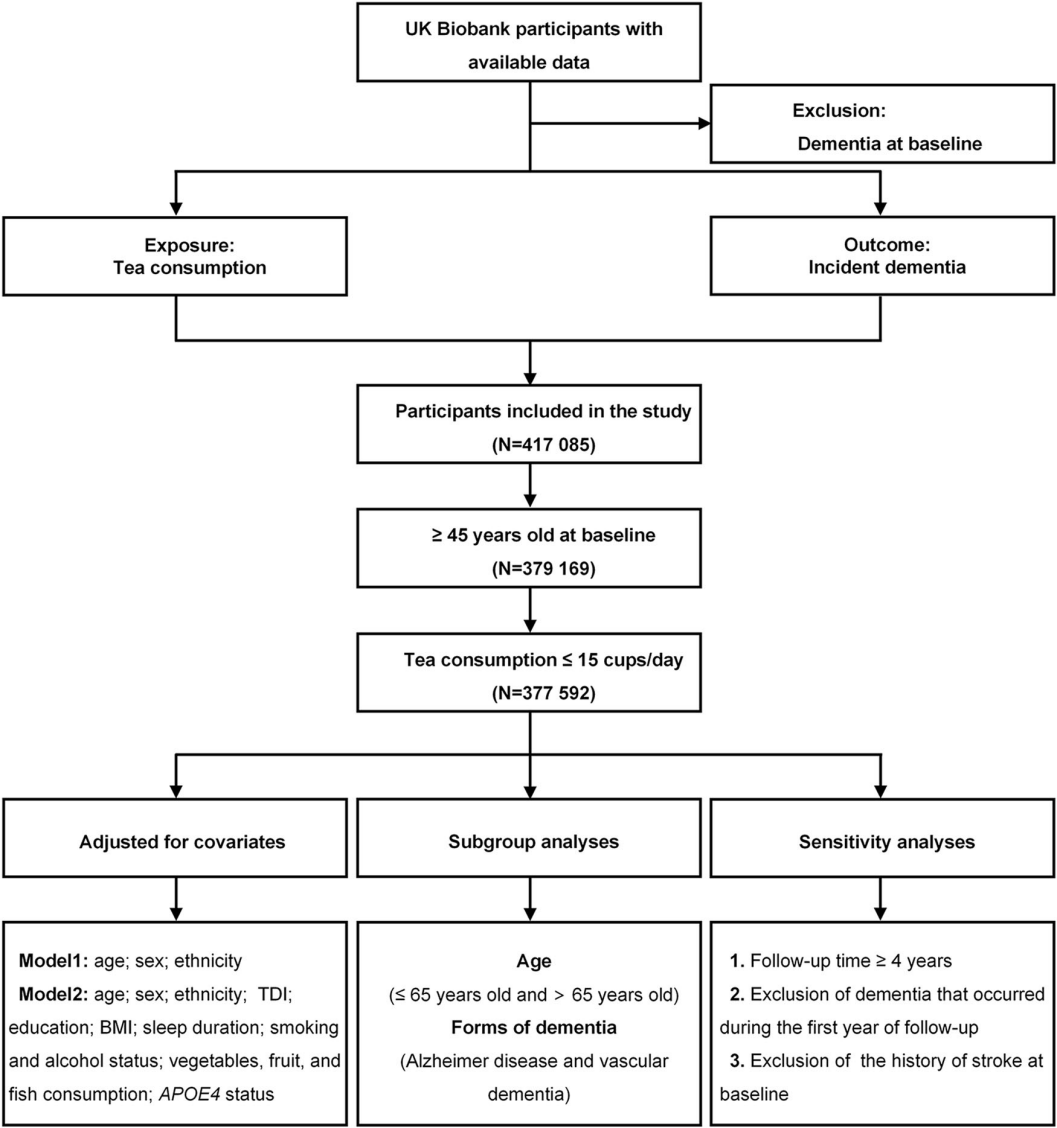
Flowchart of participant selection. TDI Townsend deprivation index, BMI Body mass index, APOE4 *apolipoprotein E4*.

At baseline, a total of 377,592 participants from the primary care dataset were included in this study (Fig. 1). Their mean age was 58.49 (SD 6.83) years; 204,980 participants (54.3%) were women. Around 85.1% of the participants reported consuming tea. During a median follow-up of 9.09 years (interquartile range [IQR] 7.08–10.64), 5122 incident dementia events were recorded. Baseline characteristics of participants stratified by the occurrence of dementia were shown in Table 1, and the missing data for certain variables were recorded in Supplementary Table 1. Demented cases were older, more likely to be *APOE4* carriers, more economically deprived, less educated, more likely to smoke, and less likely to be addicted to alcohol. Demented cases also had higher BMIs, longer sleep, and more frequent consumption of vegetables, fruit as well as fish. More males than females were diagnosed with dementia in the study population. However, there was no significant difference in the consumption of tea between demented cases and non-demented participants (the proportion of non-drinkers 14.8% vs. 15.5%, *P* = 0.163). Participant characteristics across six categories according to the daily consumption of tea were shown in Supplementary Table 2. In addition, characteristics of participants stratified by age or sex and characteristics of participants stratified by the occurrence of AD or VD were shown in Supplementary Tables 3, 4, and 5, respectively.

**Table 1:** Baseline characteristics of participants stratified by the occurrence of dementia.

Variables	All participants (*n* = 377,592)	No incident dementia (*n* = 372,470)	Incident dementia (*n* = 5122)	*P*-value
**Age (mean** ± **SD), years**	58.49 ± 6.83	58.41 ± 6.82	64.55 ± 4.39	<0.001^b^
**Age group (years),** ***n*** **(%)**				
Midlife	311,107 (82.4)	308,598 (82.9)	2509 (49.0)	–
Late-life	66,485 (17.6)	63,872 (17.1)	2613 (51.0)	–
**Female,** ***n*** **(%)**	204,980 (54.3)	202,580 (54.3)	2400 (46.8)	<0.001^c^
**White,** ***n*** **(%)**	358,908 (95.1)	354,036 (95.0)	4872 (95.1)	0.823^c^
**Whether reported consuming tea,** ***n*** **(%)**				0.163^c^
Non-consumption, *n* (%)	55,935 (14.8)	55,141 (14.8)	794 (15.5)	
Consumption, *n* (%)	321,657 (85.1)	317,329 (85.1)	4328 (84.4)	
***APOE4*** **carrier status, where reported**^**a**^, ***n*** **(%)**				<0.001^c^
Carrier	93,049 (28.5)	9075 (28.2)	1974 (45.2)	–
Non-carrier	233,914 (71.5)	231,523 (71.8)	2391 (54.8)	–
**Townsend deprivation index (median, IQR), where reported**^**a**^	−2.20 (−3.67, 0.43)	−2.20 (−3.67, 0.42)	1.89 (−3.52, 1.31)	<0.001^d^
**Education, where reported**^**a**^, ***n*** **(%)**				<0.001^c^
Without college degree	184,074 (62.0)	181,946 (62.0)	2128 (68.4)	–
With college degree	112,722 (38.0)	111,741 (38.0)	981 (31.6)	–
**Body mass index (mean** ± **SD), kg/m**^**2**^**, where reported**^**a**^	27.60 ± 4.81	27.60 ± 4.81	27.81 ± 4.95	0.002^b^
**Typical sleep duration (mean** ± **SD), hours, where reported**^**a**^	7.16 ± 1.13	7.16 ± 1.13	7.29 ± 1.38	<0.001^b^
**Smoking status, where reported**^**a**^, ***n*** **(%)**				<0.001^c^
Current	38,360 (10.2)	37,817 (10.2)	543 (10.7)	–
Previous	137,212 (36.5)	135,018 (36.4)	2194 (43.2)	–
Never	200,486 (53.3)	198,140 (53.4)	2346 (46.2)	–
**Alcohol status, where reported**^**a**^, ***n*** **(%)**				<0.001^c^
Current	345,956 (91.7)	341,592 (91.8)	4364 (85.4)	–
Previous	14,316 (3.8)	13,944 (3.7)	372 (7.3)	–
Never	16,882 (4.5)	16,508 (4.4)	374 (7.3)	–
**Vegetable consumption (median, IQR), times/ week, where reported**^**a**^	4 (3, 6)	4 (3, 6)	5 (3, 7)	<0.001^d^
**Fruit consumption (median, IQR), times/ week, where reported**^**a**^	3 (2, 4)	3 (2, 4)	3 (2, 5)	<0.001^d^
**Fish consumption (median, IQR), times/ week, where reported**^**a**^	2 (1, 3.5)	2 (1, 3.5)	2 (1.5, 4)	<0.001^d^

^a^Some patients had missing data for these variables. The missing data were not reported here.

^b^Comparisons between groups were performed via the t-test, ^c^chi-square test, ^d^Mann-Whitney U-test.

*SD* Standardized deviation, *IQR* Interquartile range, *APOE4 apolipoprotein E4.*

### Association between tea consumption and dementia

**Fig. 2:**
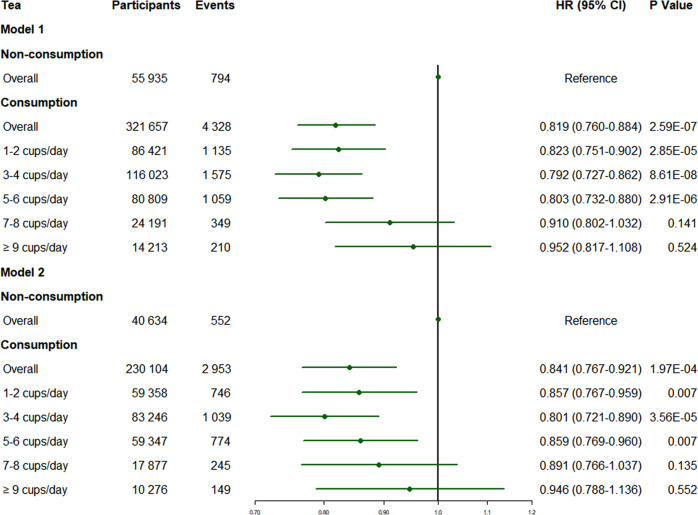
Association between tea consumption and the risk of incident dementia. In both adjusted models, tea consumption was associated with a reduced risk of dementia (model 1: HR = 0.819 [95% CI:0.760-0.884]; model 2: HR = 0.841 [95% CI: 0.767–0.921]). Moderate consumption (1–6 cups/day) of tea exerted significant protective effects. *P* values were computed by Cox proportional hazard regression. Model 1 was adjusted for age, sex and ethnicity. Model 2 was adjusted for age, sex, ethnicity, TDI, education, BMI, typical sleep duration, smoking status, alcohol status, total consumption of vegetables, total consumption of fruit, total consumption of fish and *APOE4* status. HR Hazard ratios, CI Confidence interval, TDI Townsend deprivation index, BMI Body mass index, *APOE4, apolipoprotein E4*.

Results from Cox proportional hazard regression models were shown in Supplementary Table 6 and Fig. 2. There was a significant association between tea consumption and a decreased risk of incident dementia after controlling for age, sex and ethnicity (model 1). Even in the further-adjusted model (model 2), the protective effect remained significant. Generally, HRs for incident dementia were 0.819 (95% CI: 0.760–0.884) in model 1 and 0.841 (95% CI: 0.767–0.921) in model 2 for those who reported consumption of tea compared with the non-drinkers. As for different categories of tea consumption, participants with moderate tea consumption (1–6 cups/day) had a lower hazard of dementia compared with the non-drinkers, with the specific results as follows: model 1, HR = 0.823 (95% CI:0.751–0.902) for 1–2 cups/day, HR = 0.792 (95% CI: 0.727–0.862) for 3–4cups/day, HR = 0.803 (95% CI: 0.732–0.880) for 5–6 cups/day; model 2, HR = 0.857 (95% CI: 0.767–0.959) for 1–2 cups/day, HR = 0.801 (95% CI: 0.721–0.890) for 3–4 cups/day, HR = 0.859 (95% CI: 0.769–0.960) for 5–6 cups/day. However, no significant difference was found in dementia risk between individuals who consumed more than 6 cups of tea per day and non-drinkers, with the specific results as follows: model 1, HR = 0.910 (95% CI: 0.802–1.032) for 7–8 cups/day, HR = 0.952 (95% CI: 0.817–1.108) for ≥9 cups/day; model 2, HR = 0.891 (95% CI: 0.766–1.037) for 7–8 cups/day, HR = 0.946 (95% CI: 0.788–1.136) for ≥9 cups/day.

Results of subgroup analyses stratified by age were shown in Supplementary Table 7. In midlife, there were significant associations of tea consumption with a reduced risk of dementia in both two models, and the dementia incidence was approximately 17–26% lower among individuals with tea consumption of 1–6 cups per day in the fully-adjusted model. However, this association disappeared in the late-life after controlling for all covariates (see Supplementary Fig. 1).

Results of subgroup analyses stratified by sex were shown in Supplementary Table 8. Females who consumed 3–4 cups of tea a day were less likely to develop dementia in both two models (HR = 0.800 [95% CI: 0.687–0.933] in the fully-adjusted model). More pronounced associations were found in males, and the consumption of 1–6 cups/day led to a 19 to 22% reduction in dementia incidence in the fully-adjusted model (see Supplementary Fig. 2).

**Fig. 3:**
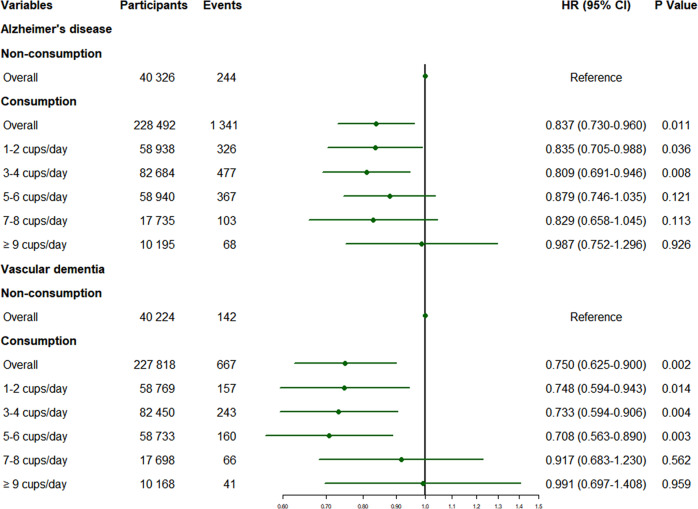
Associations of tea consumption with risks of AD and VD in the fully-adjusted model. After adjusting for all covariates, participants who consumed 1–4 cups of tea a day were 16–19% less likely to develop AD compared with non-drinkers. Besides, the tea consumption of 1–6 cups/day brought a 25 to 29% reduction in VD incidence. *P* values were computed by Cox proportional hazard regressions. Covariates included age, sex, ethnicity, TDI, education, BMI, typical sleep duration, smoking status, alcohol status, total consumption of vegetables, total consumption of fruit, total consumption of fish and *APOE4* status. HR Hazard ratios, CI Confidence interval, AD Alzheimer disease, VD Vascular dementia, TDI Townsend deprivation index, BMI Body mass index, *APOE4 apolipoprotein E4*.

Results from the subgroup analyses stratified by the form of dementia (AD/VD) were summarized in Supplementary Table 9. In model 1, the consumption of 1–6 cups/day was associated with a lower incidence of AD. In model 2, participants who consumed 1–4 cups of tea a day were 16 to 19% less likely to develop AD, but the HR for 5–6 cups/day was not significant (Fig. 3). In terms of incident VD, a similar picture to ACD was seen in both adjusted models. Moderate consumption (1–6 cups/day) led to a 25 to 29% reduction in VD incidence (Fig. 3).

### Non-linear relationship between tea consumption and dementia

**Fig. 4:**
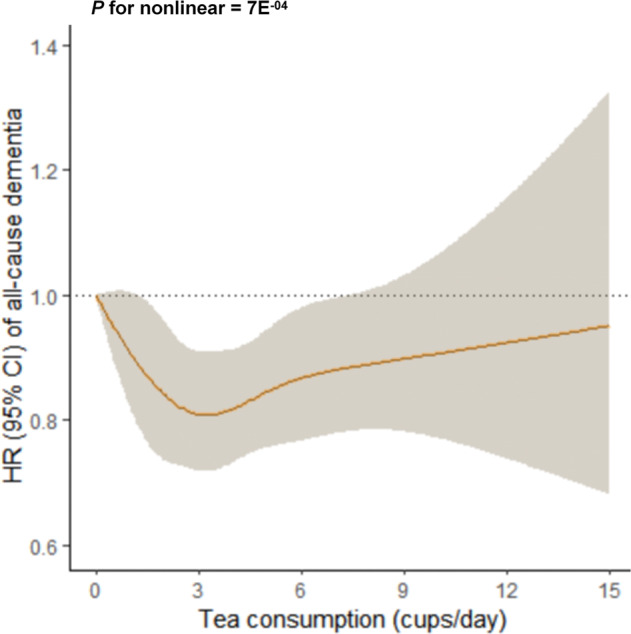
A non-linear relationship between tea consumption and incident dementia. There was a U-shaped association between tea consumption and incident dementia, and the consumption of around three cups per day showed the strongest protective effect (*P* for non-linearity = 7E^-04^). *P-*value was computed using restricted cubic splines functions in the Cox proportional hazard regression model. The model was adjusted for age, sex, ethnicity, TDI, education, BMI, typical sleep duration, smoking status, alcohol status, total consumption of vegetables, total consumption of fruit, total consumption of fish and *APOE4* status. HR Hazard ratios, CI Confidence interval, TDI Townsend deprivation index, BMI Body mass index, *APOE4 apolipoprotein E4*.

In Fig. 4, we used restricted cubic splines to flexibly model and visualize the association of tea-consumption with dementia. After adjusting for all the covariates, the risk of incident dementia substantially decreased until it reached bottom at around 3 cups/day, and increased thereafter (*P* for non-linearity = 7E^−04^). Within 3 cups/day, the HR for dementia was 0.943 (95% CI: 0.907–0.981) per cup increase, indicating an approximately 6% reduced risk for one extra cup a day.

A similar U-shaped relationship was observed in midlife, and the risk of dementia reached bottom at around 3 cups of tea per day (*P* for non-linearity = 8E^−04^, Supplementary Fig. 3A). We didn’t observe any nonlinear relationship in the late-life (see Supplementary Fig. 3B). The non-linear relationships were observed in both females and males. The largest reduction of dementia incidence was associated with intake of around 3 cups per day for females (*P* for non-linearity = 0.006, Supplementary Fig. 4A) and intakes of 3–6 cups per day for males (*P* for non-linearity = 0.022, Supplementary Fig. 4B). Furthermore, the U-shaped associations of tea-consumption with AD and VD were shown, and the largest reductions in both incidences were associated with tea consumption of 2 to 3 cups per day (AD: *P* for non-linearity = 0.046, Supplementary Fig. 5A; VD: *P* for non-linearity = 0.019, Supplementary Fig. 5B).

### Sensitivity analyses

When we restricted participants to those with a follow-up time of ≥4 years (*n* = 355 114), the HRs were similar to the main results in both of the adjusted models (see Supplementary Table 10). Sensitivity analyses after excluding dementia cases that occurred within the first 1 year follow-up (*n* = 57) to control the reverse causality showed that the HRs were of similar magnitude to the main results (see Supplementary Table 11). Exclusion of participants who had a history of stroke at baseline (*n* = 11 643) also did not change these associations significantly (see Supplementary Table 12).

## Discussion

Leveraging data from 377 592 individuals from the UKB, this study investigated the association between tea consumption and dementia over the 9-year follow-up. Our results showed that moderate drinkers (1–6 cups/day) were less likely to develop ACD, AD, and VD after adjustment for covariates. Another major finding was the U-shaped association of tea consumption with dementia risk, and the optimal intake was around 3 cups per day. These findings suggested tea to be a modifiable lifestyle factor for dementia prevention.

Previous studies yielded inconsistent results. A study among 9375 Chinese older adults showed that participants who consumed <2 cups/day, 2–4 cups/day, and ≥4 cups/day were less likely to have cognitive impairment compared with non-drinkers, which supported our findings; however, the design was cross-sectional and the reference selection was different from our analyses [14]. A longitudinal study among 1305 older adults also found that tea consumption could reduce the risk of cognitive decline compared with non-consumption over 5.3 years of follow-up [32]. The Ohsaki Cohort Study among 13,645 Japanese found that green tea consumption was associated with a lower risk of incident dementia over 5.7 years of follow-up, which was consistent with our major finding [26]. In addition, Zhang et al. conducted a large-scale cohort study and found that tea consumption was associated with lower risks of dementia as well as VD [29]. However, with tea consumption at low frequency (≤1 times per week) as the reference, a longitudinal study found no significant association of frequent tea consumption (≥3 times per week) with AD [28]. The insignificant association probably resulted from the small sample size (81 AD cases among 1836 individuals) and excessive category combination. By the way, although inconsistent results were shown regarding coffee intake, most of the studies showed a reduction for the risk of dementia [33–35]. It should be noted that tea and coffee shared some molecules (e.g., polyphenols and caffeine) which are considered to reduce dementia risk. Compared with previous researches, this cohort study used a larger sample of non-demented older adults. Sufficient cases of ACD, AD and VD were observed during a long follow-up time. Our study focused on the dosage-dependent associations of tea consumption with ACD, AD and VD, obtaining nonlinear relationships between daily dosage and dementia incidences. Compared to Zhang et al.’s, our study considered *APOE4* status as a covariate in the full-adjusted model and focused on AD in addition to VD. The age range of the total participants (≥45 years old) was larger in this study, and the effect modification by age or sex was observed in subgroup analyses. When calculating the relationships between categories of tea consumption and dementia risk, we used more specific category combinations, and thus the dosage-dependent relationships were more convinced and were in accordance with the results of non-linear analyses. Besides, we performed a great deal of sensitivity analyses to examine the stability of results.

Although the mechanisms underlying the associations between tea consumption and dementia are unclear, some possibilities may explain these findings. Firstly, oxidative stress was suggested to be involved in the pathogenesis of both AD and VD [36]. Recent vivo and vitro studies have focused on the antioxidant effects of tea biomolecules [37, 38]. For example, EGCG could chelate the bivalent metal-ions and prevent oxidation resulting from reactive hydroxyl radicals [39]. EGCG was also found to scavenge the free radicals, and thus alleviating the neuronal apoptosis as well as promoting the neuronal differentiation [37, 38, 40–42]. Methylxanthines, a group of biomolecules derived from tea (especially black tea), included theophylline (1,3-dimethylxanthine) and caffeine. Caffeine was suggested to have significant antioxidant effects [43, 44]. In human clinical trials, an increase in plasma antioxidant capacity after moderate intakes of green or black tea was observed [8, 30]. The antioxidative function might be a key factor in the neuroprotective role of tea in brain diseases and cognitive performance. Secondly, neuroinflammation has been demonstrated to play an important role in the progression of dementia [45]. Animal studies found that EGCG could decrease the expression of pro-inflammatory cytokines (e.g., interleukin [IL]-1β and tumor necrosis factor [TNF]-α), increase the expression of anti-inflammatory cytokines (e.g., IL-10), inhibit astrocyte activation and promote microglial activation. Methylxanthines were also found to increase the production of cerebrospinal fluid (CSF) as well as promote the clearance of neurotoxins, which might attenuate neuroinflammation and thereby provide biological plausibility for the prevention of dementia [20, 21]. Thirdly, l-theanine, a specific amino acid extracted from tea leaves, was suggested to be an intervention for cognitive improvement [46]. In a randomized controlled trial (RCT) study, electroencephalograms showed that participants treated by l-theanine had higher levels of theta waves, interpreted to be an indicator of cognitive alertness, in the temporal, frontal, parietal, and occipital lobes after 3 h of reading compared with the placebo group [47]. Noteworthily, since the beneficial compounds in tea are small, they could cross the blood-brain barrier to reach the brain parenchyma [48, 49].

Amyloid precursor protein (APP) could play an important role in the deposition of Aβ, thus accelerating AD progression [50]. Noteworthily, tea polyphenols including epicatechin (EC) and epigallocatechin (EGC) were found to suppress APP processing by neutralizing excessively free iron via chelation reactions [51]. Besides, AD mouse models have showed reduced Aβ pathology in brain after a long-term oral delivery of tea polyphenols [16–18]. Furthermore, previous studies in cognitively normal individuals showed that the components of green tea could alleviate abnormal tau metabolism and mitigate the detrimental effects of tau pathology, suggesting green tea as a potential prophylactic for AD [15].

The association of green or black tea with VD was partly due to their protection against transient ischaemic attack (TIA) and stroke, as suggested by population-based studies [23, 52, 53]. Besides, the protective effects of tea on VD might relate to its positive impacts on cerebral blood vessels. For example, tea was suggested to have vasodilative functions by enhancing the releases of prostacyclin and NO, as well as reducing oxidative stress [54]. Accumulating evidence has shown that tea consumption could reduce blood pressure as well as serum lipid levels, improving cerebral perfusion and reducing the risk of VD [55–58]. Moreover, caffeine in tea was revealed to protect against ischemic neuronal injury by blocking the receptors of neurotransmitter adenosine [59–61]. However, VD represents a heterogeneous group of dementia with different pathophysiologies depending on the source (e.g., small vessel disease versus large ischemia) and also shares some pathologies with AD [62]. Therefore, advances in the classification of dementia subtypes may help estimate the associations more consistently in the future.

Additionally, our study revealed a non-linear association of tea consumption with dementia and suggested daily consumption of three cups to be strongest protective. Within three cups, the benefits of tea consumption increased with daily dosage. Previous studies on the dosage-dependent relationship between tea consumption and dementia were scarce. A dose-response meta-analysis found that one cup of tea per day led to a 6% reduction in the risk of dementia or mild cognitive impairment (MCI), and two cups led to an 11% decrease [11]. However, the data extracted for this meta-analysis were not enough to calculate a nonlinear relationship convincingly, because only three studies were included, and two of them didn’t take the daily dosage of tea into consideration. Importantly, as our results showed, increase in consumption beyond 3 cups/day was not associated with increased risk of harm, but the magnitude of the benefit was reduced, and the estimates did not reach significance when daily consumption was beyond 6 cups. One possible explanation was that excessive caffeine in tea could disturb sleep and block the anti-stress effects in humans [63, 64]. Also, a study on caffeinated drinks showed that excessive intake of caffeine (>6 cups of drinks per day) might cause competitive binding to adenosine receptors and thus cause the morphological changes in brain [65]. Moreover, estimates from higher intakes included smaller numbers of participants, and this could result in the imprecision observed for dementia at these levels of tea consumption. Anyhow, our analyses indicated that the best intervention effect might be achieved at around three cups of tea per day in a future RCT study, and this intervention would not result in significant harm to participants.

More pronounced associations were observed in midlife in this study. The associations in the late-life were attenuated possibly due to confounding factors. Confounders such as BMI, alcohol intake and dietary habits were associated with both tea consumption and dementia, but these relations may differ in magnitude and even direction between midlife and the late-life. Most importantly, the pathological processes leading to dementia start many years before the clinical manifestation of cognitive decline, and the longitudinal changes related to tea consumption in the brain may also begin earlier in adult life. A population-based study found tea could mitigate AD pathologies in individuals aged 65 years or younger, which was consistent with our findings [15]. Therefore, tea consumption may be listed as one of the strategies during midlife to prevent dementia. Moreover, males seemed to benefited more from tea consumption than females in this study. Previous evidence showed that brain structure and function differed between males and females throughout the aging process, and the sex differences might have an influence on the protective effects of tea [66]. Previous cross-sectional studies found that tea consumption had a protective effect against amnestic MCI in males rather than females [67, 68]. Frequent tea consumption was found to mitigate tau pathologies in males, but not in females [15]. In the UKB, males were more likely to be tea drinkers, and this could also make the protective effects of tea more pronounced. The sex difference in the effects of tea highlighted the importance of differential preventive measures in meles and females.

This study has several strengths. We had a large sample size. In addition, we used two degrees of confounder-adjustment in the analyses to make the results more convincing. The results after adjusting for more extensive confounders (including education, TDI, BMI, lifestyle factors, dietary factors and *APOE4* status) were stable. Besides, we discussed the potential mechanisms underlying the effects of tea on brain actions in greater depth than previous studies. This study also has some limitations. Firstly, the dynamic changes in tea consumption during follow-up might influence the dosage-dependent associations. However, tea consumption was only reported at baseline in this study. Secondly, we only had information on tea consumption by cup measures, but no universal standard tea cup size was recognized. Therefore, the optimal consumption for preventing dementia identified in our analyses should be regarded as an estimation. Thirdly, the kinds and concentrations of bioactive components varied among the processing methods of tea (such as black and green tea) [69]. However, there was no information about the type of tea, limiting our ability to examine the biomolecule-related mechanisms. Studies exploring the associations between different types of tea and dementia are warranted in the future.

In conclusion, tea consumption was shown to be associated with a lower risk of dementia in the total participants. As a potentially modifiable lifestyle factor, tea consumption could play a pivotal role in the primary prevention for dementia. This study would offer a relatively simple and low-cost solution to the interventions of age-related cognitive decline or dementia.

## Supplementary information

Supplementary files

## Data Availability

The datasets described in this manuscript are available from the UK Biobank with an approved protocol. External investigators can request the data and approval of use on application to the UK Biobank (www.ukbiobank.ac.uk/).

## References

[1] Organization WH: Dementia fact Sheet. *[Internet] Available from*: http://www.hoint/mediacentre/factsheets/fs362/en/ (accessed 2 September 2021).

[2] Malik R, Georgakis MK, Neitzel J, Rannikmäe K, Ewers M, Seshadri S (2021). Midlife vascular risk factors and risk of incident dementia: Longitudinal cohort and Mendelian randomization analyses in the UK Biobank. Alzheimer’s Dement: J Alzheimer’s Assoc.

[3] Livingston G, Huntley J, Sommerlad A, Ames D, Ballard C, Banerjee S (2020). Dementia prevention, intervention, and care: 2020 report of the Lancet Commission. Lancet (Lond, Engl).

[4] Zhang H, Greenwood DC, Risch HA, Bunce D, Hardie LJ, Cade JE (2021). Meat consumption and risk of incident dementia: cohort study of 493,888 UK Biobank participants. Am J Clin Nutr.

[5] Ros E. Can specific nutrients, foods, or dietary patterns modulate cognitive function in (older) adults? Latest evidence from randomized controlled trials. Current opinion in clinical nutrition and metabolic care. 2021;24:511–20. 10.1097/MCO.0000000000000795 34596061

[6] Kivipelto M, Mangialasche F, Ngandu T (2018). Lifestyle interventions to prevent cognitive impairment, dementia and Alzheimer disease. Nat Rev Neurol.

[7] Bell IR (2005). Diet and nutrition in Alzheimer’s disease and other dementias of late life. Explor (N. Y, NY).

[8] Cabrera C, Artacho R, Giménez R (2006). Beneficial effects of green tea-a review. J Am Coll Nutr.

[9] Shin S, Lee JE, Loftfield E, Shu XO, Abe SK, Rahman MS et al. Coffee and tea consumption and mortality from all causes, cardiovascular disease and cancer: A pooled analysis of prospective studies from the Asia Cohort Consortium. Int. J. Epidemiol (in the press). 10.1093/ije/dyab161 (2021). 10.1093/ije/dyab161 PMC9308394 34468722

[10] Keller A, Wallace TC (2021). Tea intake and cardiovascular disease: an umbrella review. Ann Med.

[11] Ran LS, Liu WH, Fang YY, Xu SB, Li J, Luo X (2021). Alcohol, coffee and tea intake and the risk of cognitive deficits: a dose-response meta-analysis. Epidemiol Psychiatr Sci.

[12] Yasmeen H, Hasnain S (2015). In vitro antioxidant effect of Camellia sinensis on human cell cultures. Pak J Pharm Sci.

[13] Mancini E, Beglinger C, Drewe J, Zanchi D, Lang UE, Borgwardt S (2017). Green tea effects on cognition, mood and human brain function: A systematic review. Phytomedicine: Int J Phytother Phytopharmacol.

[14] Shen W, Xiao Y, Ying X, Li S, Zhai Y, Shang X (2015). Tea consumption and cognitive impairment: A cross-sectional study among Chinese elderly. PloS one.

[15] Ma YH, Wu JH, Xu W, Shen XN, Wang HF, Hou XH (2020). Associations of green tea consumption and cerebrospinal fluid biomarkers of Alzheimer’s Disease pathology in cognitively intact older adults: The CABLE study. J Alzheimer’s Dis: JAD.

[16] Cox CJ, Choudhry F, Peacey E, Perkinton MS, Richardson JC, Howlett DR (2015). Dietary (-)-epicatechin as a potent inhibitor of βγ-secretase amyloid precursor protein processing. Neurobiol aging.

[17] Zeng YQ, Wang YJ, Zhou XF (2014). Effects of (-)epicatechin on the pathology of APP/PS1 transgenic mice. Front Neurol.

[18] Zhang Z, Wu H, Huang H (2016). Epicatechin plus treadmill exercise are neuroprotective against moderate-stage amyloid precursor Protein/Presenilin 1 mice. Pharmacogn Mag.

[19] Serafini M, Ghiselli A, Ferro-Luzzi A (1996). In vivo antioxidant effect of green and black tea in man. Eur J Clin Nutr.

[20] Ohta A, Sitkovsky M. Methylxanthines, inflammation, and cancer: fundamental mechanisms. Handbook Exp Pharmacol. 2011;(200):469–81. 10.1007/978-3-642-13443-2_19 20859809

[21] Han ME, Kim HJ, Lee YS, Kim DH, Choi JT, Pan CS (2009). Regulation of cerebrospinal fluid production by caffeine consumption. BMC Neurosci.

[22] Schimidt HL, Vieira A, Altermann C, Martins A, Sosa P, Santos FW (2014). Memory deficits and oxidative stress in cerebral ischemia-reperfusion: neuroprotective role of physical exercise and green tea supplementation. Neurobiol Learn Mem.

[23] Shen L, Song LG, Ma H, Jin CN, Wang JA, Xiang MX (2012). Tea consumption and risk of stroke: A dose-response meta-analysis of prospective studies. J Zhejiang Univ Sci B.

[24] Arendash GW, Schleif W, Rezai-Zadeh K, Jackson EK, Zacharia LC, Cracchiolo JR (2006). Caffeine protects Alzheimer’s mice against cognitive impairment and reduces brain beta-amyloid production. Neuroscience.

[25] Chu YF, Chang WH, Black RM, Liu JR, Sompol P, Chen Y (2012). Crude caffeine reduces memory impairment and amyloid β(1-42) levels in an Alzheimer’s mouse model. Food Chem.

[26] Tomata Y, Sugiyama K, Kaiho Y, Honkura K, Watanabe T, Zhang S (2016). Green Tea Consumption and the Risk of Incident Dementia in Elderly Japanese: The Ohsaki Cohort 2006 Study. Am J Geriatr Psychiatry: Off J Am Assoc Geriatr Psychiatry.

[27] Kitamura K, Watanabe Y, Nakamura K, Sanpei K, Wakasugi M, Yokoseki A (2016). Modifiable Factors Associated with Cognitive Impairment in 1,143 Japanese Outpatients: The Project in Sado for Total Health (PROST). Dement Geriatr Cogn Disord Extra.

[28] Dai Q, Borenstein AR, Wu Y, Jackson JC, Larson EB (2006). Fruit and vegetable juices and Alzheimer’s disease: the Kame Project. Am J Med.

[29] Zhang Y, Yang H, Li S, Li WD, Wang Y (2021). Consumption of coffee and tea and risk of developing stroke, dementia, and poststroke dementia: A cohort study in the UK Biobank. PLoS Med.

[30] Rietveld A, Wiseman S (2003). Antioxidant effects of tea: evidence from human clinical trials. J Nutr.

[31] R Core Team: R: A Language and Environment for Statistical Computing. In. R Foundation for Statistical Computing, Vienna, Austria. URL https://www.R-project.org/; 2021.

[32] Shirai Y, Kuriki K, Otsuka R, Kato Y, Nishita Y, Tange C (2020). Green tea and coffee intake and risk of cognitive decline in older adults: the National Institute for Longevity Sciences, Longitudinal Study of Aging. Public Health Nutr.

[33] Eskelinen MH, Ngandu T, Tuomilehto J, Soininen H, Kivipelto M (2009). Midlife coffee and tea drinking and the risk of late-life dementia: a population-based CAIDE study. J Alzheimer’s Dis: JAD.

[34] Eskelinen MH, Kivipelto M (2010). Caffeine as a protective factor in dementia and Alzheimer’s disease. J Alzheimer’s Dis: JAD.

[35] van Gelder BM, Buijsse B, Tijhuis M, Kalmijn S, Giampaoli S, Nissinen A (2007). Coffee consumption is inversely associated with cognitive decline in elderly European men: the FINE Study. Eur J Clin Nutr.

[36] Luca M, Luca A, Calandra C (2015). The role of oxidative damage in the pathogenesis and progression of Alzheimer’s Disease and vascular Dementia. Oxid Med Cell Longev.

[37] Pervin M, Unno K, Ohishi T, Tanabe H, Miyoshi N, Nakamura Y. Beneficial effects of green tea catechins on neurodegenerative diseases. Molecules (Basel, Switzerland). 2018;23:1297. 10.3390/molecules23061297 PMC6099654 29843466

[38] Singh NA, Mandal AK, Khan ZA (2016). Potential neuroprotective properties of epigallocatechin-3-gallate (EGCG). Nutr J.

[39] Kumamoto M, Sonda T, Nagayama K, Tabata M (2001). Effects of pH and metal ions on antioxidative activities of catechins. Biosci, Biotechnol, Biochem.

[40] Soung HS, Wang MH, Tseng HC, Fang HW, Chang KC (2015). (-)Epigallocatechin-3-gallate decreases the stress-induced impairment of learning and memory in rats. Neurosci Lett.

[41] Unno K, Nakamura Y. Green tea suppresses brain aging. Molecules (Basel, Switzerland). 2021;26:4897. 10.3390/molecules26164897 PMC8401650 34443485

[42] Rodríguez-Morató J, Xicota L, Fitó M, Farré M, Dierssen M, de la Torre R (2015). Potential role of olive oil phenolic compounds in the prevention of neurodegenerative diseases. Molecules (Basel, Switz).

[43] Poole R, Kennedy OJ, Roderick P, Fallowfield JA, Hayes PC, Parkes J (2017). Coffee consumption and health: umbrella review of meta-analyses of multiple health outcomes. BMJ (Clin Res ed).

[44] Azam S, Hadi N, Khan NU, Hadi SM (2003). Antioxidant and prooxidant properties of caffeine, theobromine and xanthine. Med Sci Monit: Int Med J Exp Clin Res.

[45] Kakutani S, Watanabe H, Murayama N. Green tea intake and risks for dementia, Alzheimer’s disease, mild cognitive impairment, and cognitive Impairment: A Systematic Review. Nutrients. 2019;11:1165. 10.3390/nu11051165 PMC6567241 31137655

[46] Lavretsky H (2016). Lifestyle medicine for prevention of cognitive decline: Focus on green tea.. Am J Geriatr Psychiatry: Off J Am Assoc Geriatr Psychiatry.

[47] Park SK, Jung IC, Lee WK, Lee YS, Park HK, Go HJ (2011). A combination of green tea extract and l-theanine improves memory and attention in subjects with mild cognitive impairment: a double-blind placebo-controlled study. J Medicinal Food.

[48] Wong AD, Ye M, Levy AF, Rothstein JD, Bergles DE, Searson PC (2013). The blood-brain barrier: An engineering perspective. Front Neuroeng.

[49] Franke H, Galla HJ, Beuckmann CT (1999). An improved low-permeability in vitro-model of the blood-brain barrier: Transport studies on retinoids, sucrose, haloperidol, caffeine and mannitol. Brain Res.

[50] Pimplikar SW, Ghosal K (2011). Amyloid precursor protein: More than just neurodegeneration. Stem cell Res Ther.

[51] Wang YJ, Thomas P, Zhong JH, Bi FF, Kosaraju S, Pollard A (2009). Consumption of grape seed extract prevents amyloid-beta deposition and attenuates inflammation in brain of an Alzheimer’s disease mouse. Neurotox Res.

[52] Chung M, Zhao N, Wang D, Shams-White M, Karlsen M, Cassidy A (2020). Dose-response relation between tea consumption and risk of cardiovascular disease and all-cause mortality: A systematic review and meta-analysis of population-based studies. Adv Nutr (Bethesda, Md).

[53] Wang M, Bai Y, Wang Z, Zhang Z, Liu D, Lian X (2021). Higher tea consumption is associated with decreased risk of small vessel stroke. Clin Nutr (Edinb, Scotl).

[54] Bogdanski P, Suliburska J, Szulinska M, Stepien M, Pupek-Musialik D, Jablecka A (2012). Green tea extract reduces blood pressure, inflammatory biomarkers, and oxidative stress and improves parameters associated with insulin resistance in obese, hypertensive patients. Nutr Res (N. Y, NY).

[55] Yarmolinsky J, Gon G, Edwards P (2015). Effect of tea on blood pressure for secondary prevention of cardiovascular disease: A systematic review and meta-analysis of randomized controlled trials. Nutr Rev.

[56] Cornelis MC, van Dam RM (2020). Habitual coffee and tea consumption and cardiometabolic biomarkers in the UK Biobank: The role of beverage types and genetic variation. J Nutr.

[57] Ninomiya T, Ohara T, Hirakawa Y, Yoshida D, Doi Y, Hata J (2011). Midlife and late-life blood pressure and dementia in Japanese elderly: The Hisayama study. Hypertension (Dallas, Tex: 1979).

[58] Emdin CA, Rothwell PM, Salimi-Khorshidi G, Kiran A, Conrad N, Callender T (2016). Blood pressure and risk of vascular dementia: Evidence from a primary care registry and a cohort study of transient ischemic attack and stroke. Stroke.

[59] Nehlig A, Daval JL, Debry G (1992). Caffeine and the central nervous system: mechanisms of action, biochemical, metabolic and psychostimulant effects. Brain Res Brain Res Rev.

[60] Lunt MJ, Ragab S, Birch AA, Schley D, Jenkinson DF (2004). Comparison of caffeine-induced changes in cerebral blood flow and middle cerebral artery blood velocity shows that caffeine reduces middle cerebral artery diameter. Physiological Meas.

[61] Schwarzschild MA, Xu K, Oztas E, Petzer JP, Castagnoli K, Castagnoli N (2003). Neuroprotection by caffeine and more specific A2A receptor antagonists in animal models of Parkinson’s disease. Neurology.

[62] Kalaria RN, Kenny RA, Ballard CG, Perry R, Ince P, Polvikoski T (2004). Towards defining the neuropathological substrates of vascular dementia. J Neurol Sci.

[63] Unno K, Noda S, Kawasaki Y, Yamada H, Morita A, Iguchi K, et al. Reduced stress and improved sleep quality caused by green tea are associated with a reduced caffeine content. Nutrients. 2017;9:777. 10.3390/nu9070777 PMC5537891 28753943

[64] Jahrami H, Al-Mutarid M, Penson PE, Al-Islam Faris M, Saif Z, Hammad L. Intake of caffeine and its association with physical and mental health status among university students in Bahrain. Foods (Basel, Switzerland). 2020;9:473. 10.3390/foods9040473 PMC7230284 32290044

[65] Pham K, Mulugeta A, Zhou A, O’Brien JT, Llewellyn DJ, Hyppönen E. High coffee consumption, brain volume and risk of dementia and stroke. Nutritional Neurosci. 2021:1–12. 10.1080/1028415X.2021.1945858 34165394

[66] Giedd JN, Raznahan A, Mills KL, Lenroot RK (2012). Review: Magnetic resonance imaging of male/female differences in human adolescent brain anatomy. Biol Sex Differences.

[67] Xu H, Wang Y, Yuan Y, Zhang X, Zuo X, Cui L (2018). Gender differences in the protective effects of green tea against amnestic mild cognitive impairment in the elderly Han population. Neuropsychiatr Dis Treat.

[68] Huang CQ, Dong BR, Zhang YL, Wu HM, Liu QX (2009). Association of cognitive impairment with smoking, alcohol consumption, tea consumption, and exercise among Chinese nonagenarians/centenarians. Cogn Behav Neurol: Off J Soc Behav Cogn Neurol.

[69] Tong W, Yu J, Wu Q, Hu L, Tabys D, Wang Y (2021). Black tea quality is highly affected during processing by its leaf surface microbiome. J Agric Food Chem.

