# Supplementary material for: Tea consumption and risk of incident dementia: A prospective cohort study of 377 592 UK Biobank participants
Source: Transl Psychiatry. 2022 Apr 26;12:171. doi: 10.1038/s41398-022-01923-z (PMC9042826; doi:10.1038/s41398-022-01923-z)
Supplement: Supplementary file 1 — Supplementary files [file 41398_2022_1923_MOESM1_ESM.docx]

**Contents**

| **TITLE** | **PAGE** |
| --- | --- |
| **Supplementary Table 1. Missing data for certain variables** | 2 |
| **Supplementary Table 2. Participant characteristics across six categories according to the daily consumption of tea** | 3 |
| **Supplementary Table 3. Characteristics of participants stratified by age** | 5 |
| **Supplementary Table 4. Characteristics of participants stratified by sex** | 7 |
| **Supplementary Table 5. Characteristics of participants stratified by the occurrence of AD or VD** | 9 |
| **Supplementary Table 6. Associations between tea consumption and incident dementia in UK Biobank** | 11 |
| **Supplementary Table 7. Age-stratified analysis of the association between tea consumption and dementia** | 12 |
| **Supplementary Figure 1. Age-stratified analysis of the association between tea consumption and dementia in the fully-adjusted model** | 13 |
| **Supplementary Table 8. Sex-stratified analysis of the association between tea consumption and dementia** | 15 |
| **Supplementary Figure 2. Sex-stratified analysis of the association between tea consumption and dementia in the fully-adjusted model** | 16 |
| **Supplementary Table 9. Risks of AD and VD in participants consuming different cups of tea** | 18 |
| **Supplementary Figure 3. A non-linear relationship between tea consumption and dementia in the subgroup analysis stratified by age** | 20 |
| **Supplementary Figure 4. A non-linear relationship between tea consumption and dementia in the subgroup analysis stratified by sex** | 21 |
| **Supplementary Figure 5. Non-linear effects of tea consumption on AD and VD** | 22 |
| **Supplementary Table 10. Risks of dementia in participants consuming different cups of tea in sensitivity analysis restricting individuals to those with the follow-up time of ≥ 4 years** | 23 |
| **Supplementary Table 11. Risks of dementia in participants consuming different cups of tea in sensitivity analysis after excluding individuals who progressed to dementia during the one-year follow-up** | 24 |
| **Supplementary Table 12. Risks of dementia in participants consuming different cups of tea in sensitivity analysis after excluding individuals with a history of stroke at baseline** | 25 |

**Supplementary Table 1. Missing data for certain variables**

| **Variables** |  | **No progression** |  | **Progression to dementia** |
| --- | --- | --- | --- | --- |
| **TDI, n (%)** |  | 430 (0.1) |  | 5 (0.1) |
| **Education ^a^, n (%)** |  | 78 783 (21.2) |  | 2 013 (39.3) |
| **BMI, n (%)** |  | 2 048 (0.5) |  | 62 (1.2) |
| **Sleep duration, n (%)** |  | 2 284 (0.6) |  | 74 (1.4) |
| **Smoking status ^a^, n (%)** |  | 1 495 (0.4) |  | 39 (0.8) |
| **Alcohol status ^a^, n (%)** |  | 426 (0.1) |  | 12 (0.2) |
| **Vegetable consumption, n (%)** |  | 27 495 (7.4) |  | 461 (9.0) |
| **Fruit consumption, n (%)** |  | 38 304 (10.3) |  | 496 (9.7) |
| **Fish consumption, n (%)** |  | 8 761 (2.4) |  | 199 (3.9) |
| ***APOE4* carrier status, n (%)** |  | 49 872 (13.4) |  | 757 (14.8) |

^a^ For these categorical variables (education, smoking and alcohol), the responses of “do not know/ prefer not to answer” were classified as a specific “unknown” category in the analyses.

Abbreviations: *APOE4*, *apolipoprotein E4*; TDI, Townsend deprivation index; BMI, Body mass index.

**Supplementary Table 2. Participant characteristics across six categories according to the daily consumption of tea**

| **Variables** |  | **Non-consumption** |  | **1-2 cups/day** |  | **3-4 cups/day** |  | **5-6 cups/day** |  | **7-8 cups/day** |  | **≥ 9 cups/day** |
| --- | --- | --- | --- | --- | --- | --- | --- | --- | --- | --- | --- | --- |
| **Participants, n (%)** |  | 55 935 (14.8) |  | 86 421 (22.8) |  | 116 023 (30.7) |  | 80 809 (21.4) |  | 24 191 (6.4) |  | 14 213 (3.7) |
| **Age (mean±SD), years** |  | 58.49±6.83 |  | 58.28±6.93 |  | 58.92±6.77 |  | 58.73±6.72 |  | 58.47±6.76 |  | 58.1±6.77 |
| **Age group (years), n (%)** |  |  |  |  |  |  |  |  |  |  |  |  |
| Midlife |  | 47 679 (85.2) |  | 71 546 (82.8) |  | 93 787 (80.8) |  | 66 115 (81.8) |  | 19 975 (82.6) |  | 12 005 (84.5) |
| Late-life |  | 8 256 (14.8) |  | 14 875 (17.2) |  | 22 236 (19.2) |  | 14 694 (18.2) |  | 4 216 (17.4) |  | 2 208 (15.5) |
| **Female, n (%)** |  | 31 661 (56.6) |  | 45 505 (52.6) |  | 63 698 (54.9) |  | 43 952 (54.3) |  | 13 349 (55.1) |  | 6 815 (47.9) |
| **White, n (%)** |  | 53 711 (96.0) |  | 79 383 (91.8) |  | 109 940 (94.7) |  | 78 433 (97.0) |  | 23 650 (97.7) |  | 13 791 (97.0) |
| ***APOE4* carrier status** ^a^**, n (%)** |  |  |  |  |  |  |  |  |  |  |  |  |
| Carrier |  | 13 803 (24.6) |  | 20 408 (28.5) |  | 28 827 (28.7) |  | 20 586 (28.6) |  | 6 289 (29.0) |  | 3 553 (28.4) |
| Non-carrier |  | 34 940 (62.4) |  | 51 108 (71.5) |  | 71 773 (71.3) |  | 51 291 (71.4) |  | 15 410 (71.0) |  | 8 975 (71.6) |
| **TDI (median, IQR)** ^a^ |  | -2.21 (-3.67, 0.43) |  | -2.16 (-3.66, 0.52) |  | -2.32 (-3.74, 0.15) |  | -2.30 (-3.70, 0.20) |  | -2.15 (-3.62, 0.56) |  | -1.62 (-3.40, 1.42) |
| **Education** ^a^**, n (%)** |  |  |  |  |  |  |  |  |  |  |  |  |
| Without college degree |  | 184 074 (62.0) |  | 41 411 (58.3) |  | 56 027 (61.2) |  | 39 605 (64.1) |  | 11 874 (65.5) |  | 6 614 (65.1) |
| With college degree |  | 112 722 (38.0) |  | 29 618 (41.7) |  | 35 446 (38.8) |  | 22 171 (35.9) |  | 6 262 (34.5) |  | 3 547 (34.9) |
| **BMI (mean±SD)** ^a^**, kg/m2** |  | 27.60±4.81 |  | 26.81±4.77 |  | 27.38±4.64 |  | 27.53±4.67 |  | 27.53±4.72 |  | 27.80±4.87 |
| **Sleep duration (mean±SD)** ^a^**, hours** |  | 7.16±1.13 |  | 7.15±1.11 |  | 7.18±1.10 |  | 7.17±1.12 |  | 7.16±1.16 |  | 7.12±1.31 |
| **Smoking status** ^a^**, n (%)** |  |  |  |  |  |  |  |  |  |  |  |  |
| Current |  | 38 360 (10.2) |  | 7 785 (9.0) |  | 9 019 (7.8) |  | 7 805 (9.7) |  | 3 176 (13.2) |  | 3 002 (21.2) |
| Previous |  | 137 212 (36.5) |  | 31 695 (36.8) |  | 42 457 (36.7) |  | 29 402 (36.6) |  | 8 533 (35.4) |  | 4 892 (34.6) |
| Never |  | 200 486 (53.3) |  | 46 601 (54.1) |  | 64 082 (55.5) |  | 43 232 (53.7) |  | 12 395 (51.4) |  | 6 252 (44.2) |
| **Alcohol status** ^a^**, n (%)** |  |  |  |  |  |  |  |  |  |  |  |  |
| Current |  | 345 956 (91.7) |  | 79 827 (92.5) |  | 107 589 (92.8) |  | 74 387 (92.1) |  | 21 894 (90.6) |  | 12 452 (87.8) |
| Previous |  | 14 316 (3.8) |  | 2 609 (3.0) |  | 3 508 (3.0) |  | 3 114 (3.9) |  | 1 131 (4.7) |  | 962 (6.8) |
| Never |  | 16 882 (4.5) |  | 3 869 (4.5) |  | 4 808 (4.1) |  | 3 226 (4.0) |  | 1 137 (4.7) |  | 774 (5.5) |
| **Vegetable consumption (median, IQR)** ^a^**, times/ week** |  | 4 (3, 6) |  | 4 (3, 6) |  | 4 (3, 6) |  | 4 (3, 6) |  | 4 (3, 6) |  | 4 (3, 6) |
| **Fruit consumption (median, IQR)** ^a^**, times/ week** |  | 3 (2, 4) |  | 3 (2, 4) |  | 3 (2, 4) |  | 3 (2, 4) |  | 3 (1, 4) |  | 2 (1, 4) |
| **Fish consumption (median, IQR)** ^a^**, times/ week** |  | 2 (1, 3.5) |  | 2 (1.5, 3.5) |  | 2 (1.5, 3.5) |  | 2 (1.5, 3.5) |  | 2 (1, 3.5) |  | 1.5 (1, 3.5) |

^a^ Some patients had missing data for these variables. The missing data were not reported here.

Abbreviations: SD, standardized deviation; IQR, interquartile range; *APOE4, apolipoprotein E4*; TDI, Townsend deprivation index; BMI, Body mass index*.*

**Supplementary Table 3. Characteristics of participants stratified by age**

| **Variables** |  | **Midlife**  **(n=311 107)** |  | **Late-life**  **(n=66 485)** |  | **P value** |
| --- | --- | --- | --- | --- | --- | --- |
| **Age (mean****±SD), years** |  | 56.57±5.93 |  | 67.50±1.20 |  | <0.001 ^b^ |
| **Incident dementia, n (%)** |  | 2 509 (0.8) |  | 2 613 (3.9) |  | <0.001 ^c^ |
| **Female, n (%)** |  | 171 564 (55.1) |  | 33 416 (50.2) |  | <0.001 ^c^ |
| **White, n (%)** |  | 294 730 (94.7) |  | 64 178 (96.5) |  | <0.001 ^c^ |
| **Whether reported consuming tea, n (%)** |  |  |  |  |  | <0.001 ^c^ |
| Non-consumption, n (%) |  | 47 679 (15.3) |  | 8 256 (12.4) |  | - |
| Consumption, n (%) |  | 263 428 (84.6) |  | 58 229 (87.5) |  | - |
| ***APOE4*** ^a^**, n (%)** |  |  |  |  |  | 0.003 ^c^ |
| Carrier |  | 76 946 (28.7) |  | 16 517 (28.1) |  | - |
| Non-carrier |  | 191 187 (71.3) |  | 42 310 (71.9) |  | - |
| **TDI (median, IQR)** ^a^ |  | -2.17 (-3.66, 0.50) |  | -2.34 (-3.70, 0.13) |  | <0.001 ^d^ |
| **Education** ^a^**, n (%)** |  |  |  |  |  | <0.001 ^c^ |
| Without college degree |  | 155 646 (61.3) |  | 28 428 (66.2) |  | - |
| With college degree |  | 98 182 (38.7) |  | 14 540 (33.8) |  | - |
| **BMI (mean±SD)** ^a^**, kg/m2** |  | 26.87±4.90 |  | 27.62±4.39 |  | 0.261 ^b^ |
| **Sleep duration (mean±SD)** ^a^**, hours** |  | 7.13±1.12 |  | 7.31±1.18 |  | <0.001 ^b^ |
| **Smoking status** ^a^**, n (%)** |  |  |  |  |  | <0.001 ^c^ |
| Current |  | 33 624 (10.8) |  | 4 736 (7.2) |  | - |
| Previous |  | 108 395 (35.0) |  | 28 817 (43.6) |  | - |
| Never |  | 167 955 (54.2) |  | 32 531 (49.2) |  | - |
| **Alcohol status** ^a^**, n (%)** |  |  |  |  |  | <0.001 ^c^ |
| Current |  | 286 184 (92.1) |  | 59 772 (90.0) |  | - |
| Previous |  | 11 594 (3.7) |  | 2 722 (4.1) |  | - |
| Never |  | 12 974 (4.2) |  | 3 908 (5.9) |  | - |
| **Vegetable consumption (median, IQR)** ^a^**, times/ week** |  | 4 (3, 6) |  | 5 (3, 6) |  | <0.001 ^d^ |
| **Fruit consumption (median, IQR)** ^a^**, times/ week** |  | 3 (2, 4) |  | 3 (2, 5) |  | <0.001 ^d^ |
| **Fish consumption (median, IQR)** ^a^**, times/ week** |  | 2 (1, 3.5) |  | 2 (1.5, 4) |  | <0.001 ^d^ |

^a^ Some patients had missing data for these variables. The missing data were not reported here.

Comparisons between groups were performed via the t-test ^b^, chi-square test ^c^, and Mann-Whitney U-test ^d^.

Abbreviations: SD, standardized deviation; IQR, interquartile range; *APOE4, apolipoprotein E4*; TDI, Townsend deprivation index; BMI, Body mass index*.*

**Supplementary Table 4. Characteristics of participants stratified by sex**

| **Variables** |  | **Female**  **(n=204 980)** |  | **Male**  **(n=172 612)** |  | **P value** |
| --- | --- | --- | --- | --- | --- | --- |
| **Age (mean±SD), years** |  | 58.22±6.80 |  | 58.81±6.85 |  | <0.001 ^b^ |
| **Incident dementia, n (%)** |  | 2 400 (1.1) |  | 2 722 (1.5) |  | <0.001 ^c^ |
| **White, n (%)** |  | 194 880 (95.1) |  | 164 028 (95.0) |  | 0.519 ^c^ |
| **Whether reported consuming tea, n (%)** |  |  |  |  |  | <0.001 ^c^ |
| Non-consumption, n (%) |  | 31 661 (15.4) |  | 24 274 (14.0) |  | - |
| Consumption, n (%) |  | 173319 (84.5) |  | 148 338 (85.9) |  | - |
| ***APOE4*** ^a^**, n (%)** |  |  |  |  |  | 0.723 ^c^ |
| Carrier |  | 50 408 (28.6) |  | 43 058 (28.6) |  | - |
| Non-carrier |  | 126 089 (71.4) |  | 107 408 (71.4) |  | - |
| **TDI (median, IQR)** ^a^ |  | -2.20 (-3.66, 0.37) |  | -1.33 (-3.68, 0.50) |  | 0.032 ^d^ |
| **Education** ^a^**, n (%)** |  |  |  |  |  | <0.001 ^c^ |
| Without college degree |  | 102 847 (63.7) |  | 81 227 (60.0) |  | - |
| With college degree |  | 58 581 (36.3) |  | 54 141 (40.0) |  | - |
| **BMI (mean±SD)** ^a^**, kg/m2** |  | 27.30±5.20 |  | 27.96±4.27 |  | <0.001 ^b^ |
| **Sleep duration (mean±SD)** ^a^**, hours** |  | 7.16±1.14 |  | 7.16±1.12 |  | 0.652 ^b^ |
| **Smoking status** ^a^**, n (%)** |  |  |  |  |  | <0.001 ^c^ |
| Current |  | 17 703 (8.7) |  | 20 657 (12.0) |  | - |
| Previous |  | 66 647 (32.6) |  | 70 565 (41.1) |  | - |
| Never |  | 119 822 (58.7) |  | 80 664 (46.9) |  | - |
| **Alcohol status** ^a^**, n (%)** |  |  |  |  |  | <0.001 ^c^ |
| Current |  | 184 493 (90.1) |  | 161 463 (93.6) |  | - |
| Previous |  | 7 930 (3.9) |  | 6 386 (3.7) |  | - |
| Never |  | 12 318 (6.0) |  | 4 564 (2.6) |  | - |
| **Vegetable consumption (median, IQR)** ^a^**, times/ week** |  | 5 (3, 6) |  | 4 (3, 6) |  | <0.001 ^d^ |
| **Fruit consumption (median, IQR)** ^a^**, times/ week** |  | 3 (2, 5) |  | 2 (1, 4) |  | <0.001 ^d^ |
| **Fish consumption (median, IQR)** ^a^**, times/ week** |  | 2 (1.5, 3.5) |  | 2 (1, 3.5) |  | <0.001 ^d^ |

^a^ Some patients had missing data for these variables. The missing data were not reported here.

Comparisons between groups were performed via the t-test ^b^, chi-square test ^c^, and Mann-Whitney U-test ^d^.

Abbreviations: SD, standardized deviation; IQR, interquartile range; *APOE4, apolipoprotein E4*; TDI, Townsend deprivation index; BMI, Body mass index*.*

**Supplementary Table 5.** **Characteristics of participants stratified by the occurrence of AD or VD**

| **Variables** |  | **No incident dementia**  **(n=372 470)** |  | **AD**  **(n=2 261)** | **P value** |  | **VD**  **(n=1 231)** | **P value** |
| --- | --- | --- | --- | --- | --- | --- | --- | --- |
| **Age (mean±SD), years** |  | 58.41±6.82 |  | 64.73±4.14 | <0.001 ^b^ |  | 64.88±4.06 | <0.001 ^b^ |
| **Age group (years), n (%)** |  |  |  |  | - |  |  | - |
| Midlife |  | 308 598 (82.9) |  | 1 085 (48.0) | - |  | 568 (46.1) | - |
| Late-life |  | 63 872 (17.1) |  | 1 176 (52.0) | - |  | 663 (53.9) | - |
| **Female, n (%)** |  | 202 580 (54.3) |  | 1 176 (52.0) | 0.024 ^c^ |  | 496 (40.2) | <0.001 ^c^ |
| **White, n (%)** |  | 354 036 (95.0) |  | 2 164 (95.7) | 0.150 ^c^ |  | 1 167 (94.8) | 0.678 ^c^ |
| **Whether reported consuming tea, n (%)** |  |  |  |  | 0.625 ^c^ |  |  | 0.001 ^c^ |
| Non-consumption |  | 55 141 (14.8) |  | 343 (15.1) | - |  | 222 (18) | - |
| Consumption |  | 317 329 (85.1) |  | 1 918 (84.8) | - |  | 1 009 (81.9) | - |
| ***APOE4*** ^a^**, n (%)** |  |  |  |  | <0.001 ^c^ |  |  | <0.001 ^c^ |
| Carrier |  | 91 075 (28.2) |  | 1 232 (63.2) | - |  | 523 (48.7) | - |
| Non-carrier |  | 231 523 (71.8) |  | 717 (37.8) | - |  | 551 (51.3) | - |
| **TDI (median, IQR)** ^a^ |  | -2.20 (-3.67, 0.42) |  | -2.03 (-3.62, 1.00) | 0.003 ^d^ |  | -1.79 (-3.40, 1.62) | <0.001 ^d^ |
| **Education** ^a^**, n (%)** |  |  |  |  | 0.003 ^c^ |  |  | <0.001 ^c^ |
| Without college degree |  | 181 946 (62.0) |  | 947 (69.1) | - |  | 507 (73.5) | - |
| With college degree |  | 111 741 (38.0) |  | 423 (30.9) | - |  | 183 (26.5) | - |
| **BMI (mean±SD)** ^a^**, kg/m2** |  | 27.60±4.81 |  | 27.28±4.70 | 0.002 ^b^ |  | 28.58±5.09 | 0.007 ^b^ |
| **Sleep duration (mean±SD)** ^a^**, hours** |  | 7.16±1.13 |  | 7.30±1.29 | <0.001 ^b^ |  | 7.27±1.47 | <0.001 ^b^ |
| **Smoking status** ^a^**, n (%)** |  |  |  |  | <0.001 ^c^ |  |  | <0.001 ^c^ |
| Current |  | 37 817 (10.2) |  | 202 (9.0) | - |  | 147 (12.7) | - |
| Previous |  | 135 018 (36.4) |  | 944 (42.2) | - |  | 564 (46.5) | - |
| Never |  | 198 140 (53.4) |  | 1 092 (48.8) | - |  | 502 (41.4) | - |
| **Alcohol status** ^a^**, n (%)** |  |  |  |  | <0.001 ^c^ |  |  | <0.001 ^c^ |
| Current |  | 341 592 (91.8) |  | 1 937 (85.9) | - |  | 1 029 (83.9) | - |
| Previous |  | 13 944 (3.7) |  | 140 (6.2) | - |  | 115 (9.4) | - |
| Never |  | 16 508 (4.4) |  | 177 (7.9) | - |  | 83 (6.8) | - |
| **Vegetable consumption (median, IQR)** ^a^**, times/ week** |  | 4 (3, 6) |  | 5 (3, 7) | <0.001 ^d^ |  | 5 (3, 7) | 0.004 ^d^ |
| **Fruit consumption (median, IQR)** ^a^**, times/ week** |  | 3 (2, 4) |  | 3 (2, 5) | <0.001 ^d^ |  | 3 (2, 5) | <0.001 ^d^ |
| **Fish consumption (median, IQR)** ^a^**, times/ week** |  | 2 (1, 3.5) |  | 2 (1.5, 4) | <0.001 ^d^ |  | 2 (1.5, 4) | <0.001 ^d^ |

^a^ Some patients had missing data for these variables. The missing data were not reported here.

Comparisons between groups were performed via the t-test ^b^, chi-square test ^c^, and Mann-Whitney U-test ^d^.

Abbreviations: AD, Alzheimer's disease; VD, vascular dementia; SD, standardized deviation; IQR, interquartile range; *APOE4, apolipoprotein E4*; TDI, Townsend deprivation index; BMI, Body mass index*.*

**Supplementary Table 6. Associations between tea consumption and incident dementia in UK Biobank**

| **Tea** | **Model 1** | | | |  | **Model 2** | | | |
| --- | --- | --- | --- | --- | --- | --- | --- | --- | --- |
|  | **Participants** | **Events** | **HR (95% CI)** | ***P* value** |  | **Participants** | **Events** | **HR (95% CI)** | ***P* value** |
| Non-consumption | 55 935 | 794 | 1 (reference) |  |  | 40 634 | 552 | 1 (reference) |  |
| Consumption | 321 657 | 4 328 | 0.819 (0.760-0.884) | **2.59E-07** |  | 230 104 | 2 953 | 0.841 (0.767-0.921) | **1.97E-04** |
| 1-2 cups/day | 86 421 | 1 135 | 0.823 (0.751-0.902) | **2.85E-05** |  | 59 358 | 746 | 0.857 (0.767-0.959) | **0.007** |
| 3-4 cups/day | 116 023 | 1 575 | 0.792 (0.727-0.862) | **8.61E-08** |  | 83 246 | 1 039 | 0.801 (0.721-0.890) | **3.56E-05** |
| 5-6 cups/day | 80 809 | 1 059 | 0.803 (0.732-0.880) | **2.91E-06** |  | 59 347 | 774 | 0.859 (0.769-0.960) | **0.007** |
| 7-8 cups/day | 24 191 | 349 | 0.910 (0.802-1.032) | 0.141 |  | 17 877 | 245 | 0.891 (0.766-1.037) | 0.135 |
| ≥ 9 cups/day | 14 213 | 210 | 0.952 (0.817-1.108) | 0.524 |  | 10 276 | 149 | 0.946 (0.788-1.136) | 0.552 |
| P for trend |  |  |  | 0.092 |  |  |  |  | 0.118 |

HRs and 95% CIs for dementia according to tea consumption were calculated using Cox proportional hazard regression.

Model 1 was adjusted for age, sex and ethnicity.

Model 2 was adjusted for age, sex, ethnicity, TDI, education, BMI, typical sleep duration, smoking status, alcohol status, total consumption of vegetables, total consumption of fruit, total consumption of fish and *APOE4* status.

Abbreviations: HR, hazard ratios; CI, confidence interval; TDI, Townsend deprivation index; BMI, body mass index; *APOE4*, *apolipoprotein E4*.

**Supplementary Table 7. Age-stratified analysis of the association between tea consumption and dementia**

| **Tea** | **Model 1** | | | |  | **Model 2** | | | |
| --- | --- | --- | --- | --- | --- | --- | --- | --- | --- |
|  | **Participants** | **Events** | **HR (95% CI)** | ***P* value** |  | **Participants** | **Events** | **HR (95% CI)** | ***P* value** |
| **Midlife** |  |  |  |  |  |  |  |  |  |
| Non-consumption | 47 679 | 438 | 1 (reference) |  |  | 34 474 | 305 | 1 (reference) |  |
| Consumption | 263 428 | 2 071 | 0.774 (0.698-0.858) | **1.15e-06** |  | 187 377 | 1 395 | 0.777 (0.686-0.880) | **7.43E-05** |
| 1-2 cups/day | 71 546 | 536 | 0.752 (0.662-0.854) | **1.09e-05** |  | 48 765 | 346 | 0.753 (0.644-0.880) | **3.77E-04** |
| 3-4 cups/day | 93 787 | 730 | 0.745 (0.662-0.839) | **1.17e-06** |  | 66 975 | 476 | 0.738 (0.637-0.854) | **4.68E-05** |
| 5-6 cups/day | 66 115 | 521 | 0.771 (0.679-0.876) | **6.36e-05** |  | 48 300 | 381 | 0.831 (0.714-0.968) | **0.017** |
| 7-8 cups/day | 19 975 | 169 | 0.860 (0.720-1.027) | 0.097 |  | 14 704 | 117 | 0.813 (0.656-1.008) | 0.059 |
| ≥ 9 cups/day | 12 005 | 115 | 0.946 (0.770-1.162) | 0.596 |  | 8 633 | 75 | 0.817 (0.633-1.056) | 0.122 |
| **Late-life** |  |  |  |  |  |  |  |  |  |
| Non-consumption | 8 256 | 356 | 1 (reference) |  |  | 6 160 | 247 | 1 (reference) |  |
| Consumption | 58 229 | 2 257 | 0.874 (0.782-0.978) | **0.019** |  | 42 727 | 1 558 | 0.919 (0.803-1.052) | 0.223 |
| 1-2 cups/day | 14 875 | 599 | 0.907 (0.795-1.035) | 0.146 |  | 10 593 | 400 | 0.988 (0.840-1.162) | 0.883 |
| 3-4 cups/day | 22 236 | 845 | 0.846 (0.747-0.958) | **0.008** |  | 16 271 | 563 | 0.875 (0.752-1.019) | 0.085 |
| 5-6 cups/day | 14 694 | 538 | 0.840 (0.735-0.961) | **0.011** |  | 11 047 | 393 | 0.896 (0.763-1.053) | 0.182 |
| 7-8 cups/day | 4 216 | 180 | 0.970 (0.811-1.161) | 0.742 |  | 3 173 | 128 | 0.985 (0.794-1.222) | 0.892 |
| ≥ 9 cups/day | 2 208 | 95 | 0.960 (0.765-1.204) | 0.721 |  | 1 643 | 74 | 1.099 (0.845-1.430) | 0.481 |

HRs and 95% CIs for dementia according to tea consumption were calculated using Cox proportional hazard regression.

Model 1 was adjusted for age, sex and ethnicity. Model 2 was adjusted for age, sex, ethnicity, TDI, education, BMI, typical sleep duration, smoking status, alcohol status, total consumption of vegetables, total consumption of fruit, total consumption of fish and *APOE4* status.

Abbreviations: HR, hazard ratios; CI, confidence interval; TDI, Townsend deprivation index; BMI, body mass index; *APOE4*, *apolipoprotein E4*.

**Supplementary Figure 1. Age-stratified analysis of the association between tea consumption and dementia in the fully-adjusted model**

**
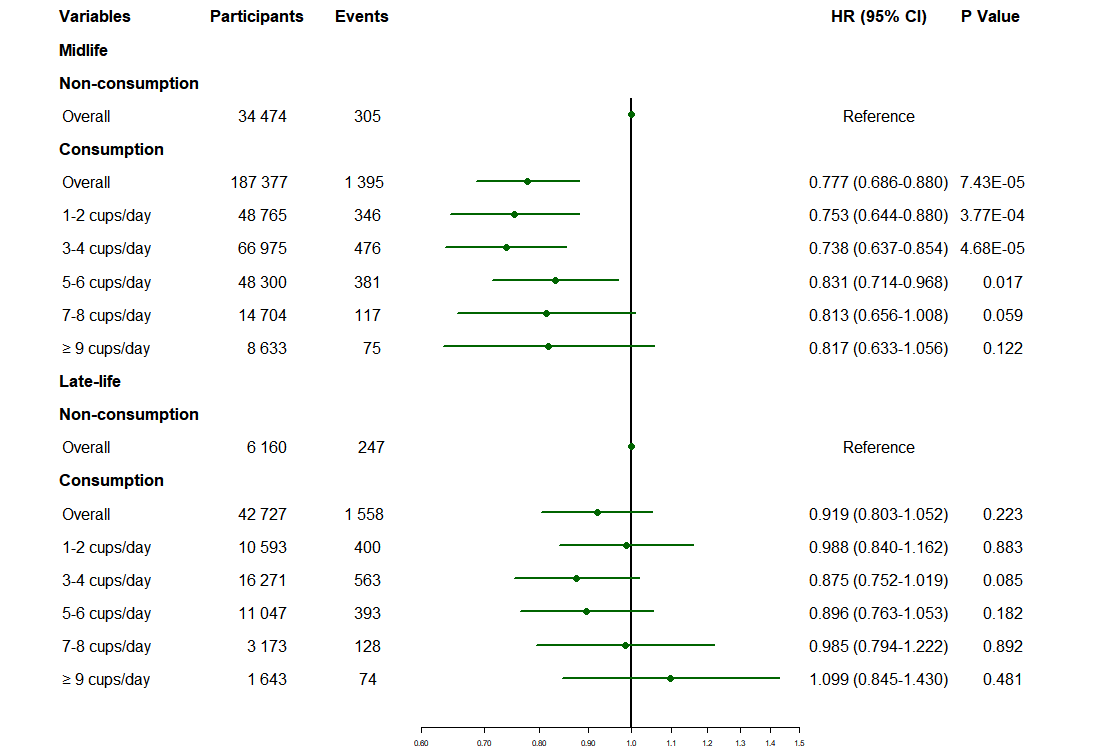
**

After accounting for all covariates, the dementia incidence was approximately 17-26% lower with the intake of 1-6 cups of tea per day in midlife. This association disappeared in the late-life.

HRs and 95% CIs for dementia according to tea consumption were calculated using Cox proportional hazard regression.

Covariates included age, sex, ethnicity, TDI, education, BMI, typical sleep duration, smoking status, alcohol status, total consumption of vegetables, total consumption of fruit, total consumption of fish and *APOE4* status.

Abbreviations: HR, hazard ratios; CI, confidence interval; TDI, Townsend deprivation index; BMI, body mass index; *APOE4*, *apolipoprotein E4*.

**Supplementary Table 8.** **Sex-stratified analysis of the association between tea consumption and dementia**

| **Tea** | **Model 1** | | | |  | **Model 2** | | | |
| --- | --- | --- | --- | --- | --- | --- | --- | --- | --- |
|  | **Participants** | **Events** | **HR (95% CI)** | ***P* value** |  | **Participants** | **Events** | **HR (95% CI)** | ***P* value** |
| **Female** |  |  |  |  |  |  |  |  |  |
| Non-consumption | 31 661 | 378 | 1 (reference) |  |  | 23 213 | 268 | 1 (reference) |  |
| Consumption | 173 319 | 2 022 | 0.872 (0.781-0.973) | **0.015** |  | 125 164 | 1 386 | 0.886 (0.776-1.010) | 0.071 |
| 1-2 cups/day | 45 505 | 529 | 0.893 (0.782-1.020) | 0.095 |  | 31 339 | 346 | 0.925 (0.786-1.088) | 0.347 |
| 3-4 cups/day | 63 698 | 702 | 0.795 (0.701-0.901) | **3.28E-04** |  | 46 194 | 468 | 0.800 (0.687-0.933) | **0.004** |
| 5-6 cups/day | 43 952 | 527 | 0.894 (0.783-1.020) | 0.095 |  | 32 658 | 391 | 0.955 (0.816-1.117) | 0.563 |
| 7-8 cups/day | 13 349 | 169 | 0.972 (0.810-1.165) | 0.755 |  | 9 959 | 116 | 0.921 (0.740-1.147) | 0.463 |
| ≥ 9 cups/day | 6 815 | 95 | 1.105 (0.882-1.383) | 0.387 |  | 5 014 | 65 | 1.056 (0.804-1.388) | 0.695 |
| **Male** |  |  |  |  |  |  |  |  |  |
| Non-consumption | 24 274 | 416 | 1 (reference) |  |  | 17 421 | 284 | 1 (reference) |  |
| Consumption | 148 338 | 2 306 | 0.773 (0.697-0.859) | **1.43****E-06** |  | 104 940 | 1 567 | 0.804 (0.708-0.913) | **7.51****E-04** |
| 1-2 cups/day | 40 916 | 606 | 0.765 (0.675-0.867) | **2.84E-05** |  | 28 019 | 400 | 0.807 (0.691-0.942) | **0.006** |
| 3-4 cups/day | 52 325 | 873 | 0.789 (0.702-0.887) | **7.27E-05** |  | 37 052 | 571 | 0.805 (0.697-0.931) | **0.003** |
| 5-6 cups/day | 36 857 | 532 | 0.724 (0.637-0.824) | **8.68E-07** |  | 26 689 | 383 | 0.779 (0.667-0.910) | **0.002** |
| 7-8 cups/day | 10 842 | 180 | 0.857 (0.720-1.021) | 0.084 |  | 7 918 | 129 | 0.878 (0.712-1.083) | 0.224 |
| ≥ 9 cups/day | 7 398 | 115 | 0.845 (0.687-1.039) | 0.110 |  | 5 262 | 84 | 0.881 (0.689-1.126) | 0.311 |

HRs and 95% CIs for dementia according to tea consumption were calculated using Cox proportional hazard regression.

Model 1 was adjusted for age and ethnicity. Model 2 was adjusted for age, ethnicity, TDI, education, BMI, typical sleep duration, smoking status, alcohol status, total consumption of vegetables, total consumption of fruit, total consumption of fish and *APOE4* status.

Abbreviations: HR, hazard ratios; CI, confidence interval; TDI, Townsend deprivation index; BMI, body mass index; *APOE4*, *apolipoprotein E4*.

**Supplementary Figure 2. Sex-stratified analysis of the association between tea consumption and dementia in the fully-adjusted model**

**
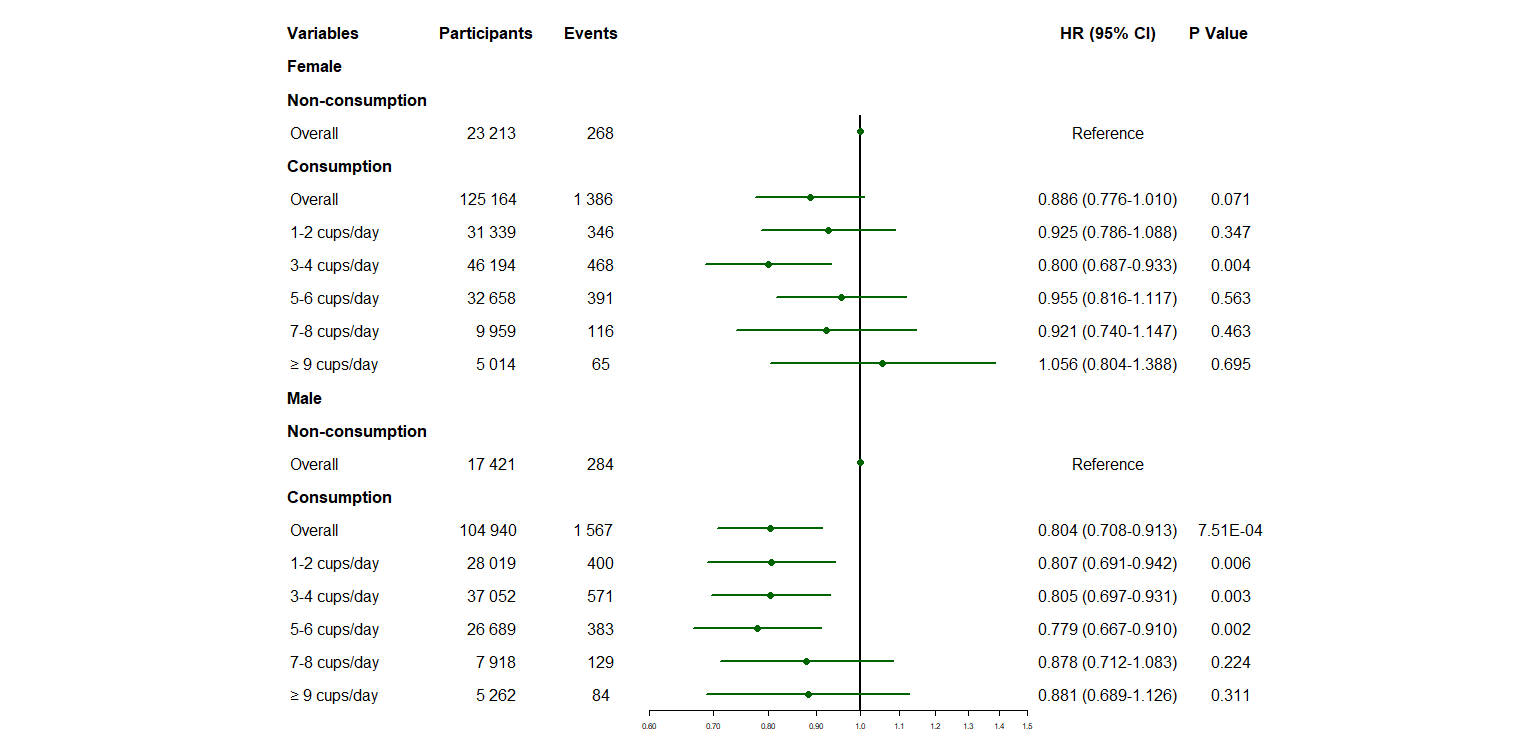
**

After accounting for all covariates, females who consumed 3-4 cups of tea a day were 20% less likely to develop dementia, and males who consumed 1-6 cups of tea a day were 19 to 22% less likely to develop dementia.

HRs and 95% CIs for dementia according to tea consumption were calculated using Cox proportional hazard regression.

Covariates included age, ethnicity, TDI, education, BMI, typical sleep duration, smoking status, alcohol status, total consumption of vegetables, total consumption of fruit, total consumption of fish and *APOE4* status.

Abbreviations: HR, hazard ratios; CI, confidence interval; TDI, Townsend deprivation index; BMI, body mass index; *APOE4*, *apolipoprotein E4*.

**Supplementary Table 9. Risks of AD and VD in participants consuming different cups of tea**

| **Tea** | **Model 1** | | | |  | **Model 2** | | | |
| --- | --- | --- | --- | --- | --- | --- | --- | --- | --- |
|  | **Participants** | **Events** | **HR (95% CI)** | ***P* value** |  | **Participants** | **Events** | **HR (95% CI)** | ***P* value** |
| **Alzheimer's disease** |  |  |  |  |  |  |  |  |  |
| Non-consumption | 55 484 | 343 | 1 (reference) |  |  | 40 326 | 244 | 1 (reference) |  |
| Consumption | 319 247 | 1 918 | 0.843 (0.751-0.946) | **0.004** |  | 228 492 | 1 341 | 0.837 (0.730-0.960) | **0.011** |
| 1-2 cups/day | 85 773 | 487 | 0.824 (0.717-0.947) | **0.006** |  | 58 938 | 326 | 0.835 (0.705-0.988) | **0.036** |
| 3-4 cups/day | 115 157 | 709 | 0.827 (0.727-0.942) | **0.004** |  | 82 684 | 477 | 0.809 (0.691-0.946) | **0.008** |
| 5-6 cups/day | 80 240 | 490 | 0.857 (0.746-0.984) | **0.028** |  | 58 940 | 367 | 0.879 (0.746-1.035) | 0.121 |
| 7-8 cups/day | 23 984 | 142 | 0.859 (0.706-1.045) | 0.127 |  | 17 735 | 103 | 0.829 (0.658-1.045) | 0.113 |
| ≥ 9 cups/day | 14 093 | 90 | 0.959 (0.760-1.211) | 0.726 |  | 10 195 | 68 | 0.987 (0.752-1.296) | 0.926 |
| **Vascular dementia** |  |  |  |  |  |  |  |  |  |
| Non-consumption | 55 363 | 222 | 1 (reference) |  |  | 40 224 | 142 | 1 (reference) |  |
| Consumption | 318 338 | 1 009 | 0.669 (0.578-0.774) | **6.07E-08** |  | 227 818 | 667 | 0.750 (0.625-0.900) | **0.002** |
| 1-2 cups/day | 85 542 | 256 | 0.644 (0.538-0.772) | **1.93E-06** |  | 58 769 | 157 | 0.748 (0.594-0.943) | **0.014** |
| 3-4 cups/day | 114 828 | 380 | 0.662 (0.561-0.782) | **1.13E-06** |  | 82 450 | 243 | 0.733 (0.594-0.906) | **0.004** |
| 5-6 cups/day | 79 975 | 225 | 0.601 (0.500-0.724) | **8.08E-08** |  | 58 733 | 160 | 0.708 (0.563-0.890) | **0.003** |
| 7-8 cups/day | 23 932 | 90 | 0.825 (0.646-1.055) | 0.125 |  | 17 698 | 66 | 0.917 (0.683-1.230) | 0.562 |
| ≥ 9 cups/day | 14 061 | 58 | 0.922 (0.690-1.232) | 0.583 |  | 10 168 | 41 | 0.991 (0.697-1.408) | 0.959 |

HRs and 95% CIs for AD or VD according to tea consumption were calculated using Cox proportional hazard regression.

Model 1 was adjusted for age, sex and ethnicity.

Model 2 was adjusted for age, sex, ethnicity, TDI, education, BMI, typical sleep duration, smoking status, alcohol status, total consumption of vegetables, total consumption of fruit, total consumption of fish and *APOE4* status.

Abbreviations: HR, hazard ratios; CI, confidence interval; AD, Alzheimer disease; VD, vascular dementia; TDI, Townsend deprivation index; BMI, body mass index; *APOE4*, *apolipoprotein E4*.

**Supplementary Figure 3. A non-linear relationship between tea consumption and dementia in the subgroup analysis stratified by age**


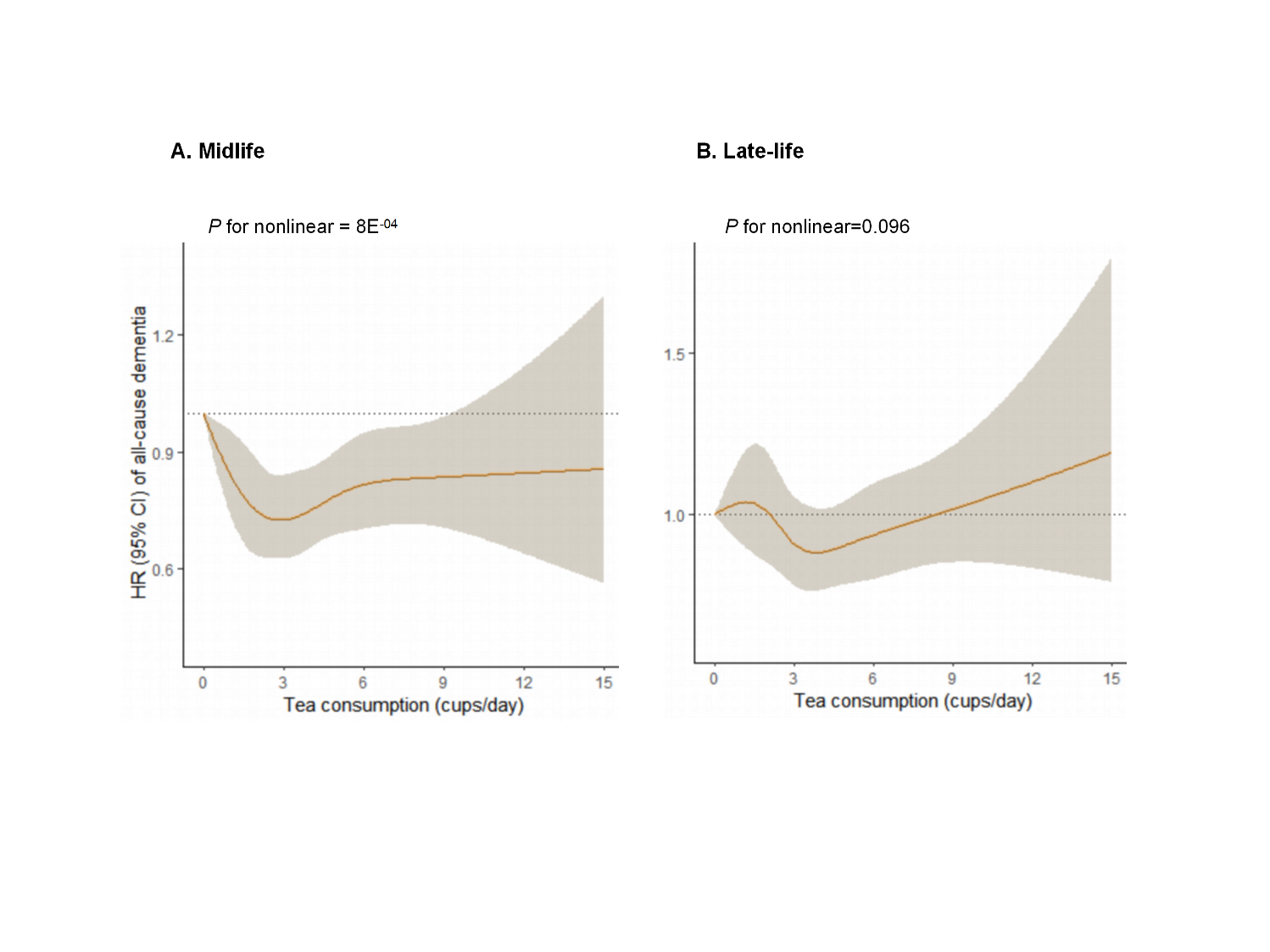


The U-shaped relation was observed among midlife, and the consumption at around three cups per day showed the most distinct protection (*P* for non-linearity = 8E^-04^) (A). There was not any nonlinear association among the late-life (*P* for non-linearity = 0.096) (B).

*P* values were computed using restricted cubic splines functions in the Cox proportional hazard regression model.

The model was adjusted for age, sex, ethnicity, TDI, education, BMI, typical sleep duration, smoking status, alcohol status, total consumption of vegetables, total consumption of fruit, total consumption of fish and *APOE4* status.

Abbreviations: HR, hazard ratios; CI, confidence interval; TDI, Townsend deprivation index; BMI, body mass index; *APOE4, apolipoprotein E4.*

**Supplementary Figure 4. A non-linear relationship between tea consumption and dementia in the subgroup analysis stratified by sex**

*
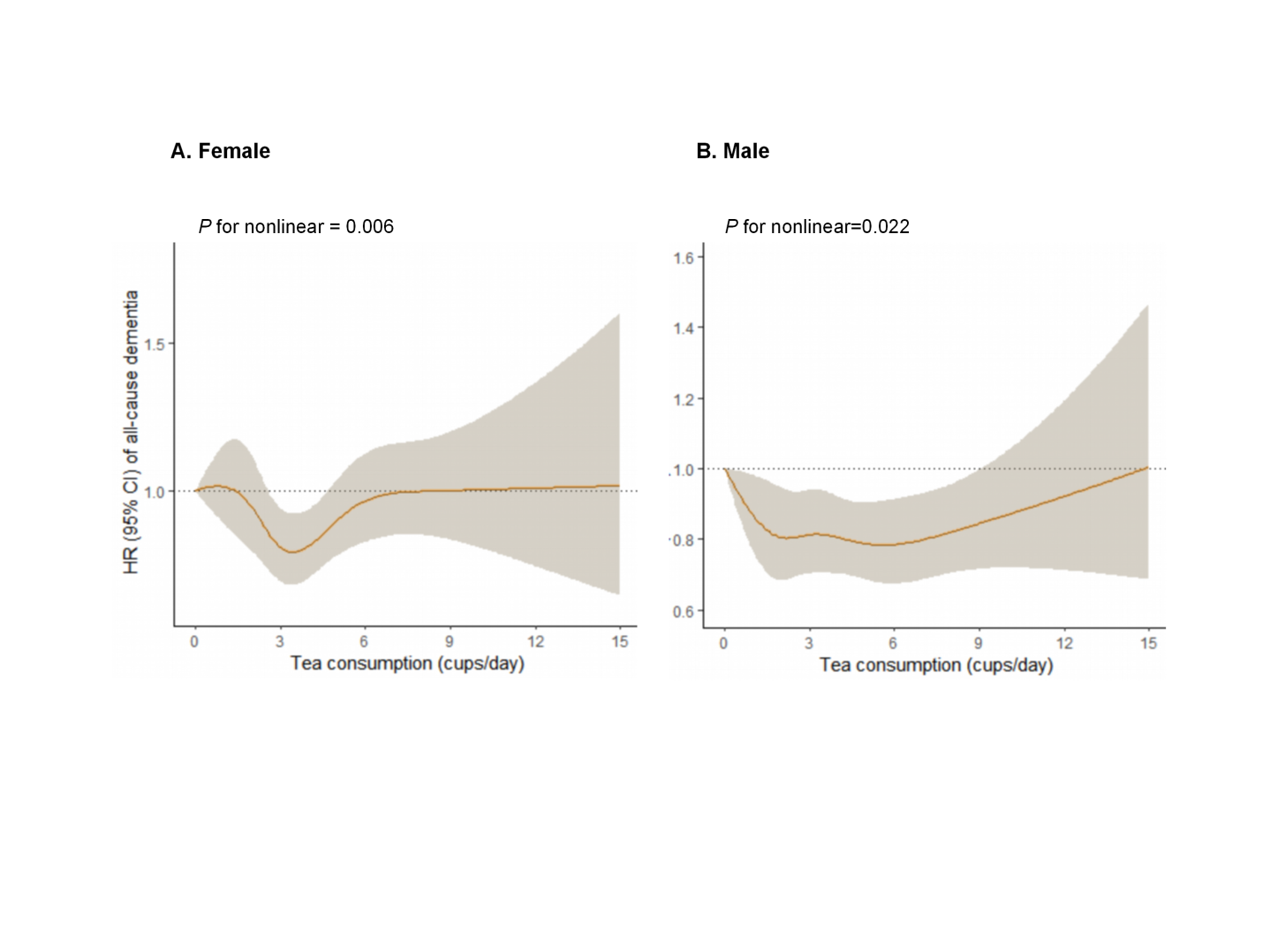
*

The non-linear relationships were observed in both females and males. The largest reduction of dementia incidence was associated with intake of 3 cups per day for females (*P* for non-linearity = 0.006) (A) and intakes of 3 to 6 cups per day for males (*P* for non-linearity = 0.022) (B).

*P* values were computed using restricted cubic splines functions in the Cox proportional hazard regression model.

The model was adjusted for age, ethnicity, TDI, education, BMI, typical sleep duration, smoking status, alcohol status, total consumption of vegetables, total consumption of fruit, total consumption of fish and *APOE4* status.

Abbreviations: HR, hazard ratios; CI, confidence interval; TDI, Townsend deprivation index; BMI, body mass index; *APOE4, apolipoprotein E4.*

**Supplementary Figure 5. Non-linear effects of tea consumption on AD and VD**


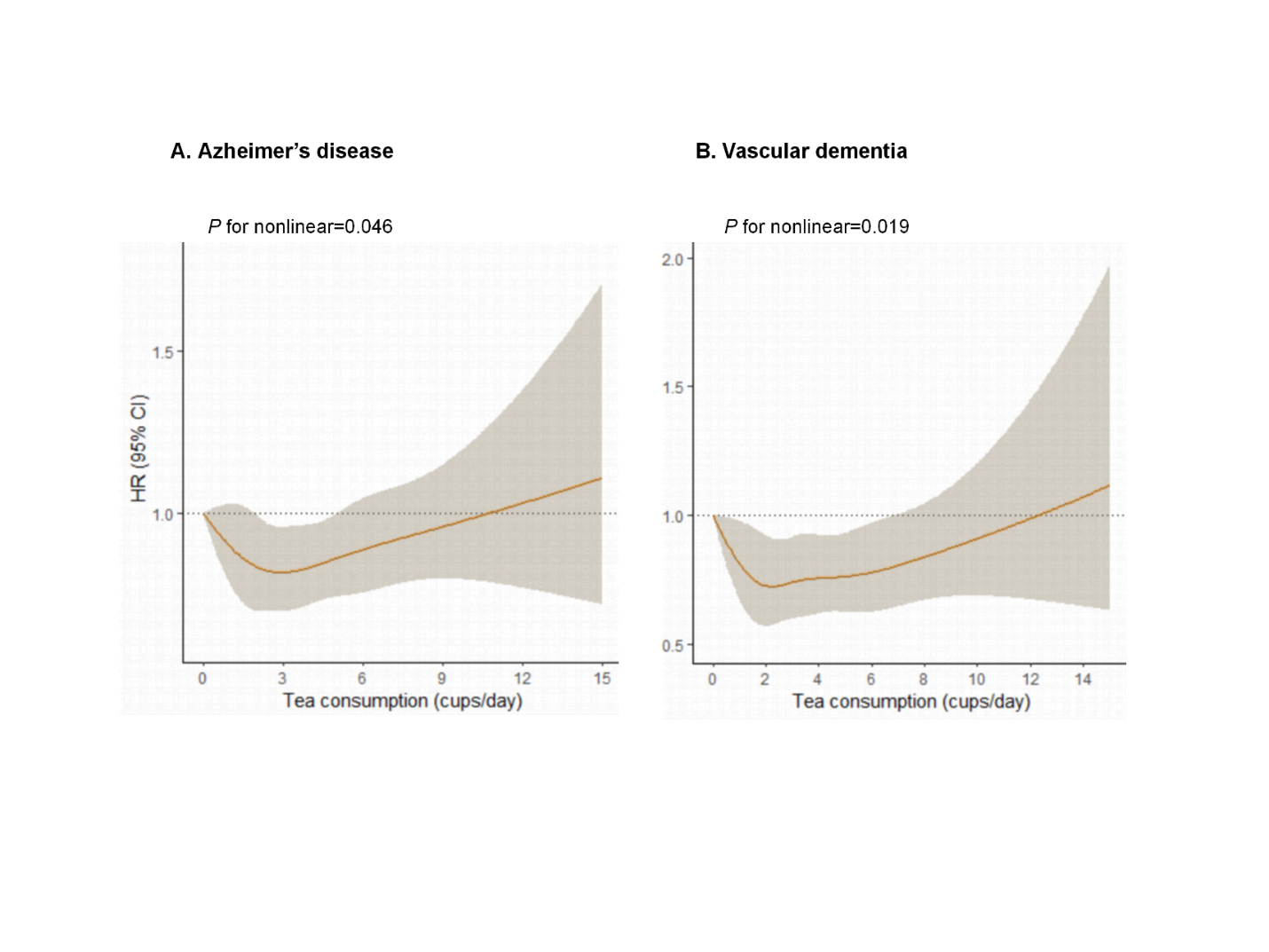


The U-shaped associations of tea-consumption with AD (*P* for non-linearity = 0.046) (A) and VD (*P* for non-linearity = 0.019) (B) were shown, and the largest reduction of incidences were associated with intakes of 2 to 3 cups per day.

*P* values were computed using restricted cubic splines functions in the Cox proportional hazard regression model.

The model was adjusted for age, sex, ethnicity, TDI, education, BMI, typical sleep duration, smoking status, alcohol status, total consumption of vegetables, total consumption of fruit, total consumption of fish and *APOE4* status.

Abbreviations: HR, hazard ratios; CI, confidence interval; AD, Alzheimer disease; VD, vascular dementia; TDI, Townsend deprivation index; BMI, body mass index; *APOE4, apolipoprotein E4.*

**Supplementary Table 10. Risks of dementia in participants consuming different cups of tea in sensitivity analysis restricting individuals to those with the follow-up time of ≥4 years**

| **Tea intake** | **Model 1** | | | |  | **Model 2** | | | |
| --- | --- | --- | --- | --- | --- | --- | --- | --- | --- |
|  | **Participants** | **Events** | **HR (95% CI)** | ***P* value** |  | **Participants** | **Events** | **HR (95% CI)** | ***P* value** |
| Non-consumption | 52 458 | 716 | 1 (reference) |  |  | 38 210 | 492 | 1 (reference) |  |
| Consumption | 302 656 | 3 903 | 0.820 (0.757-0.888) | **1.03e-06** |  | 216 961 | 2 679 | 0.857 (0.778-0.944) | **0.002** |
| 1-2 cups/day | 81 204 | 1 024 | 0.824 (0.749-0.907) | **8.10e-05** |  | 55 925 | 685 | 0.887 (0.788-0.998) | **0.046** |
| 3-4 cups/day | 109 163 | 1 419 | 0.790 (0.722-0.864) | **2.92e-07** |  | 78 451 | 931 | 0.805 (0.721-0.900) | **1.34e-04** |
| 5-6 cups/day | 761 77 | 962 | 0.809 (0.734-0.891) | **1.83e-05** |  | 56 041 | 709 | 0.887 (0.790-0.997) | **0.044** |
| 7-8 cups/day | 22 773 | 308 | 0.891 (0.779-1.019) | 0.091 |  | 16 875 | 218 | 0.894 (0.761-1.049) | 0.170 |
| ≥ 9 cups/day | 13 339 | 190 | 0.956 (0.814-1.122) | 0.581 |  | 9 669 | 136 | 0.973 (0.803-1.179) | 0.782 |

HRs and 95% CIs for dementia according to tea consumption were calculated using Cox proportional hazard regression.

Model 1 was adjusted for age, sex and ethnicity.

Model 2 was adjusted for age, sex, ethnicity, TDI, education, BMI, typical sleep duration, smoking status, alcohol status, total consumption of vegetables, total consumption of fruit, total consumption of fish and *APOE4* status.

Abbreviations: HR, hazard ratios; CI, confidence interval; TDI, Townsend deprivation index; BMI, body mass index; *APOE4*, *apolipoprotein E4*.

**Supplementary Table 11. Risks of dementia in participants consuming different cups of tea in sensitivity analysis after excluding individuals who progressed to dementia during the one-year follow-up**

| **Tea intake** | **Model 1** | | | |  | **Model 2** | | | |
| --- | --- | --- | --- | --- | --- | --- | --- | --- | --- |
|  | **Participants** | **Events** | **HR (95% CI)** | ***P* value** |  | **Participants** | **Events** | **HR (95% CI)** | ***P* value** |
| Non-consumption | 55 928 | 787 | 1 (reference) |  |  | 40 628 | 546 | 1 (reference) |  |
| Consumption | 321 607 | 4 278 | 0.817 (0.757-0.881) | **1.86e-07** |  | 230 073 | 2 922 | 0.841 (0.767-0.922) | **2.19e-04** |
| 1-2 cups/day | 86 408 | 1 122 | 0.820 (0.748-0.899) | **2.26e-05** |  | 59 351 | 739 | 0.859 (0.767-0.961) | **0.008** |
| 3-4 cups/day | 116 004 | 1 556 | 0.788 (0.723-0.859) | **5.67e-08** |  | 83 230 | 1 023 | 0.797 (0.717-0.886) | **2.60e-05** |
| 5-6 cups/day | 80 796 | 1 046 | 0.800 (0.729-0.877) | **2.26e-06** |  | 59 342 | 769 | 0.863 (0.772-0.964) | 0.009 |
| 7-8 cups/day | 24 187 | 345 | 0.907 (0.799-1.030) | **0.131** |  | 17 874 | 242 | 0.888 (0.763-1.035) | 0.128 |
| ≥ 9 cups/day | 14 212 | 209 | 0.956 (0.820-1.113) | 0.559 |  | 10 276 | 149 | 0.956 (0.796-1.148) | 0.631 |

HRs and 95% CIs for dementia according to tea consumption were calculated using Cox proportional hazard regression.

Model 1 was adjusted for age, sex and ethnicity.

Model 2 was adjusted for age, sex, ethnicity, TDI, education, BMI, typical sleep duration, smoking status, alcohol status, total consumption of vegetables, total consumption of fruit, total consumption of fish and *APOE4* status.

Abbreviations: HR, hazard ratios; CI, confidence interval; TDI, Townsend deprivation index; BMI, body mass index; *APOE4*, *apolipoprotein E4*.

**Supplementary Table 12. Risks of dementia in participants consuming different cups of tea in sensitivity analysis after excluding individuals with a history of stroke at baseline**

| **Tea intake** | **Model 1** | | | |  | **Model 2** | | | |
| --- | --- | --- | --- | --- | --- | --- | --- | --- | --- |
|  | **Participants** | **Events** | **HR (95% CI)** | ***P* value** |  | **Participants** | **Events** | **HR (95% CI)** | ***P* value** |
| Non-consumption | 53 893 | 683 | 1 (reference) |  |  | 39 174 | 481 | 1 (reference) |  |
| Consumption | 312 055 | 3 780 | 0.827 (0.762-0.897) | **4.87e-06** |  | 223 392 | 2 586 | 0.829 (0.752-0.915) | **1.83e-04** |
| 1-2 cups/day | 83 989 | 996 | 0.834 (0.756-0.919) | **2.70e-04** |  | 57 730 | 657 | 0.852 (0.756-0.961) | **0.009** |
| 3-4 cups/day | 112 670 | 1 381 | 0.801 (0.731-0.879) | **2.37e-06** |  | 80 884 | 916 | 0.794 (0.710-0.888) | **5.71e-05** |
| 5-6 cups/day | 78 454 | 929 | 0.811 (0.735-0.896) | **3.46e-05** |  | 57 668 | 678 | 0.849 (0.754-0.955) | **0.007** |
| 7-8 cups/day | 23 356 | 297 | 0.899 (0.784-1.030) | 0.125 |  | 17 276 | 210 | 0.876 (0.744-1.031) | 0.112 |
| ≥ 9 cups/day | 13 586 | 177 | 0.941 (0.798-1.111) | 0.475 |  | 9 834 | 125 | 0.917 (0.752-1.119) | 0.395 |

HRs and 95% CIs for dementia according to tea consumption were calculated using Cox proportional hazard regression.

Model 1 was adjusted for age, sex and ethnicity.

Model 2 was adjusted for age, sex, ethnicity, TDI, education, BMI, typical sleep duration, smoking status, alcohol status, total consumption of vegetables, total consumption of fruit, total consumption of fish and *APOE4* status.

Abbreviations: HR, hazard ratios; CI, confidence interval; TDI, Townsend deprivation index; BMI, body mass index; *APOE4*, *apolipoprotein E4*.
